# Conversion of walnut tyrosinase into a catechol oxidase by site directed mutagenesis

**DOI:** 10.1038/s41598-020-57671-x

**Published:** 2020-02-03

**Authors:** Felix Panis, Ioannis Kampatsikas, Aleksandar Bijelic, Annette Rompel

**Affiliations:** Universität Wien, Fakultät für Chemie, Institut für Biophysikalische Chemie, Wien, Austria

**Keywords:** Oxidoreductases, Oxidoreductases

## Abstract

Polyphenol oxidases (PPOs) comprise tyrosinases (TYRs) and catechol oxidases (COs), which catalyse the initial reactions in the biosynthesis of melanin. TYRs hydroxylate monophenolic (monophenolase activity) and oxidize diphenolic (diphenolase activity) substrates, whereas COs react only with diphenols. In order to elucidate the biochemical basis for the different reactions in PPOs, cDNA from walnut leaves was synthesized, the target gene encoding the latent walnut tyrosinase (*jr*PPO1) was cloned, and the enzyme was heterologously expressed in *Escherichia coli*. Mutations targeting the two activity controller residues (Asn240 and Leu244) as well as the gatekeeper residue (Phe260) were designed to impair monophenolase activity of *jr*PPO1. For the first time, monophenolase activity of *jr*PPO1 towards *L*-tyrosine was blocked in two double mutants (Asn240Lys/Leu244Arg and Asn240Thr/Leu244Arg) while its diphenolase activity was partially preserved, thereby converting *jr*PPO1 into a CO. Kinetic data show that recombinant *jr*PPO1 resembles the natural enzyme, and spectrophotometric investigations proved that the copper content remains unaffected by the mutations. The results presented herein provide experimental evidence that a precisely tuned interplay between the amino acids located around the active center controls the substrate specificity and therewith the mono- versus diphenolase activity in the type-III copper enzyme *jr*PPO1.

## Introduction

**Figure 1:**
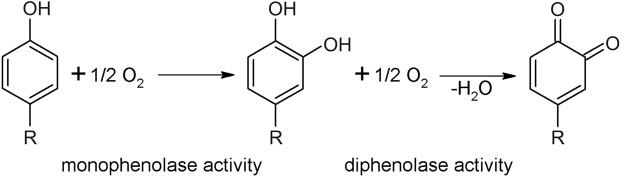
Reactions catalysed by PPOs. TYRs catalyse the *o*-hydroxylation of monophenols as well as the subsequent two electron oxidation of the resulting *o*-diphenol to the respective *o*-quinone. COs catalyse solely the oxidation of *o*-diphenols to the respective *o*-quinones.

Tyrosinases (TYRs), catechol oxidases (COs) and aurone synthases (AUSs) represent the polyphenol oxidase (PPO) family, which is an umbrella term for copper metalloenzymes^1–3^ containing one type-III copper center. TYRs catalyse the hydroxylation of monophenols to *o*-diphenols (EC 1.14.18.1, monophenolase activity) as well as the subsequent oxidation of *o*-diphenols to their corresponding *o*-quinones (EC 1.10.3.1, diphenolase activity)^1,4^, whereas COs catalyse only the latter reaction, unable to react with monophenolic substrates (Fig. 1). AUSs participate in the formation of aurones from chalcone precursors and are involved in plant secondary metabolism^5,6^. Quinones produced by PPOs usually undergo non-enzymatic reactions, polymerize^7^ and finally form melanin products^8,9^. PPOs occur in a broad spectrum of organisms, including archaea^10^, bacteria^11^, fungi^2^, plants^8^ and animals^12,13^. In plants, they are believed to be involved in defence mechanisms associated with the formation of browning substances, which is triggered by mechanical damage or wounding^14^, while in animals their reaction products are responsible for coloring of skin, hair and eyes^13^.

TYR from *Juglans regia* (walnut, *jr*PPO1) is expressed *in vivo* as a latent 66.8 kDa pro-enzyme consisting of three domains^15^: an N-terminal chloroplast transit peptide (~12 kDa)^15^, the catalytically active domain (~39 kDa) and the C-terminal domain (~16 kDa)  that shields the entrance to the catalytic pocket and keeps the enzyme in a latent state. *In vivo* enzymatic activity is triggered by the removal of the C-terminal domain^16^. Alternatively, PPOs can be activated by fatty acids^17^, acidic pH^18^, detergents, such as sodium dodecyl sulfate (SDS)^19,20^, and proteases^21^.

**Figure 2:**
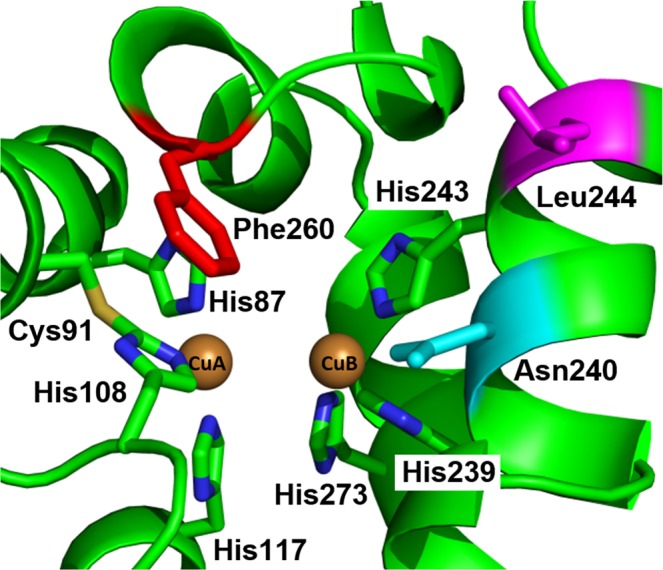
Active center of *jr*PPO1-wt (PDB entry, 5CE9). The six conserved histidines coordinating CuA (His87, His108 and His117) and CuB (His239, His243 and His273) are highlighted as sticks. Cys91 forms a conserved thioether bridge (S-C) with His108. Moreover, the gatekeeper residue Phe260 (red), the 1^st^ activity controller Asn240 (cyan) and the 2^nd^ activity controller Leu244 (magenta) are depicted as sticks.

Over the last decades, PPOs have been studied intensively in the endeavour to solve the conundrum of substrate scope. X-ray crystallography led to an increasing number of PPO structures^22–26^; however, structural comparison of TYRs (*jr*PPO1^26,27^, *Md*PPO1^16,24^ *Ab*PPO3^28^ and *Ab*PPO4^29^) and COs (*Ib*CO^22^, *Vv*PPO^23^ and *cg*AUS1^25^) revealed no considerable differences (Table S1). Walnut PPO exhibits the typical characteristics of PPOs: two copper ions (CuA and CuB) that are coordinated by three conserved histidines and are able to bind dioxygen in a side-on bridging mode (µ-η^2^:η^2^)^30^. CuA is coordinated by His87, His108 and His117, whereas CuB is coordinated by His239, His243 and His273 (Fig. 2)^26^. Moreover, the flexibility of His108 is restricted by a thioether bridge formed between the Cε atom of His108 and the sulphur atom of Cys91. More importantly, residues in close proximity to the active center have been suspected to control the activity of the enzyme^21^. The so-called gatekeeper residue, which usually features a conserved bulky phenylalanine in plant PPOs, is located inside the pocket of the active center and shields CuA^27^. On the basis of the crystal structures of *Ib*CO^22^ and *Vv*CO^23^, it was initially believed that this residue is responsible for the differentiation between mono- and diphenolase activity by restricting access to the active center^31^. However, this hypothesis was contradicted by the structure of *jr*PPO1, which also features a phenylalanine (Phe260) (Fig. 2) at the same position^26^. Therefore, it was proposed that the aromatic ring of phenylalanine plays a decisive role in substrate orientation and stabilization via *π-π-*interactions^25,26^. Based on their amino acid sequence, plant PPOs can be divided into two phylogenetic groups (group 1 and group 2), and it has been shown recently that members of group 1 exhibit mono- and diphenolase activity, whereas group 2 PPOs are only active on diphenols^32,33^. Moreover, two non-conserved amino acids were identified in apple tyrosinases (*Md*PPO1–3) and named activity controllers. These activity controllers are located next to the first (His_B1_ + 1) and second (His_B2_ + 1) CuB coordinating histidines and display a huge diversity in plant PPOs (Fig. S1). The herein investigated *jr*PPO1 has an asparagine (Asn240) as the 1^st^ activity controller and a leucine (Leu244) as the 2^nd^ activity controller (Fig. 2). These two residues are believed to influence the physicochemical properties and the redox potential of the active center and either enhance or impair the hydroxylation of monophenols by changing the ability of water molecules to deprotonate monophenolic substrates^21^.

In this study, the cloning of the *jr*PPO1 gene and the subsequent high-yield heterologous expression for biochemical experiments and site-direct mutagenesis are reported. Recombinant wild-type *jr*PPO1 (*jr*PPO1-wt) was characterized by determining the optimal SDS concentration and pH value for activity induction. Based on kinetic data (using *L*-tyrosine and *L*-DOPA as monophenolic and diphenolic substrates, respectively), recombinantly produced *jr*PPO1-wt exhibits reaction rate characteristics similar to those of the natural, purified *jr*PPO1^26,34^. Moreover, *jr*PPO1-wt was proteolytically activated with three different proteases (trypsin, proteinase K and nagarse). However, proteinase K and nagarse led to enzymatic degradation, while only the reaction with trypsin converted the enzyme to a pre-active state, as cleavage in-between the C-terminal and the active domain occurred, but the two domains remained attached to each other. Therefore,  *jr*PPO1-wt holds its latency even upon protease treatment. Cultivation of walnut trees has increased over the last decades due to the production of timber. In addition, walnut fruits offer a high nutritional value and several bioactive compounds^35,36^. Tyrosinase activity limits the availability and quality of walnut products. Hence, controlling the reactivity of *jr*PPO1 is desired by the agricultural, pharmaceutical and cosmetic industry^37,38^, as it offers an increase in nutritional values of produce and improved food quality, such as higher concentrations of antioxidants and phenolic compounds while reducing surface browning. Understanding the origin of the different reactivities in TYRs (mono- and diphenolase activity) and COs (only diphenolase activity) is a critical prerequisite for controlling PPO reactions, but it is still unclear which structural elements  trigger the monophenolase activity in some enzymes. Herein, we demonstrate that mutations applied to the activity controller residues (Asn240 and Leu244) and to the gatekeeper residue (Phe260) inhibit the monophenolase activity of *jr*PPO1 and convert the plant TYR (*jr*PPO1) into a CO. Based on the results reported in this study, we prove the significance of these three positions for the activity of *jr*PPO1. Our data profoundly contribute to the decoding of PPO activity and specifies the amino acid positions that direct mono- and diphenolase activity.

## Results and Discussion

### Cloning, heterologous expression and protein purification of *jr*PPO1-wt

**Figure 3:**
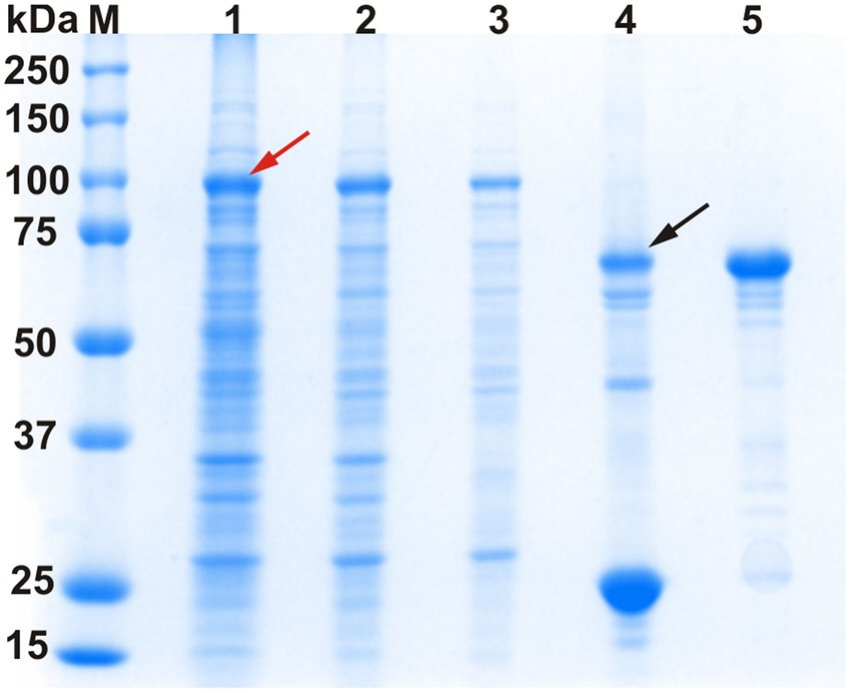
SDS-PAGE of recombinant *jr*PPO1-wt at different purification stages under reducing conditions. M = marker, lane 1: lysate (70 µg), lane 2: soluble lysate (40 µg), lane 3: eluate of the 1^st^ GSTrap column (10 µg), lane 4: GST-*jr*PPO1-wt mixed with HRV-3C-protease for 40 hours and lane 5: pure latent *jr*PPO1-wt after the 2^nd^ GSTrap column. The red arrow indicates the over-expression band of the fusion protein (GST-*jr*PPO1-wt). The black arrow shows *jr*PPO1-wt after removal of the GST-tag. The gel was cropped to display the lanes of interest (full-length gel: Fig. S23).

Total RNA was isolated from walnut leaves and cDNA was synthesized by reverse transcription. Amplification of the gene encoding the latent *jr*PPO1-wt was performed and it was cloned into the expression vector pGEX-6p-1. Sanger sequencing of the cloned gene revealed six mutations in comparison with the published *jr*PPO1 sequence (FJ769240.1)^15^, five of them were silent and one resulted in the exchange of Val107Ile, which does not influence the enzymatic behavior of *jr*PPO1 (see supporting information). The expression vector carrying the *jr*PPO1-wt gene was transformed into *E. coli* BL21 (DE3) and was expressed similar to previous studies^21,39^, resulting in a final yield of 38 mg pure protein *per* liter of culture. However, a suitable protocol is required in order to avoid production of insoluble and/or inactive PPO^40–43^. Different expression protocols have been reported, including the variation of affinity-tags, incubation temperatures and expression hosts (Table S2). However, the heterologous expression of *jr*PPO1-wt attests that the decisive factor for enhancing the yield of enzymatically active and soluble PPO is low temperature (20–26 °C), especially in combination with a glutathione-S-transferase (GST) tag^21^. The GST-tag does not only facilitate the purification but also promotes correct folding of the target protein and improves the solubility^44^. The usage of a rich medium (2xYT) in combination with a prolonged expression time (65 hours) at 20 °C led to a substantial increase in *jr*PPO1-wt yield. The induction of the expression with isopropyl-β-D-thiogalactopyranosid (IPTG, 0.5 mM) was found to result in higher yields (38 g/l) compared to autoinduction method (21 g/l) (see supporting information). Purification was performed using a two-step purification protocol (Fig. S2) in a pre-packed 5 ml GSTrap FF column (GE), leading to a high purity of at least 95% (Fig. 3). Purified *jr*PPO1-wt was stored at 4 °C in 200 mM NaCl and 50 mM Tris-HCl pH 7.8 and used immediately for kinetic measurements and activity assays.

### Proteolytic activation of *jr*PPO1-wt

**Figure 4:**
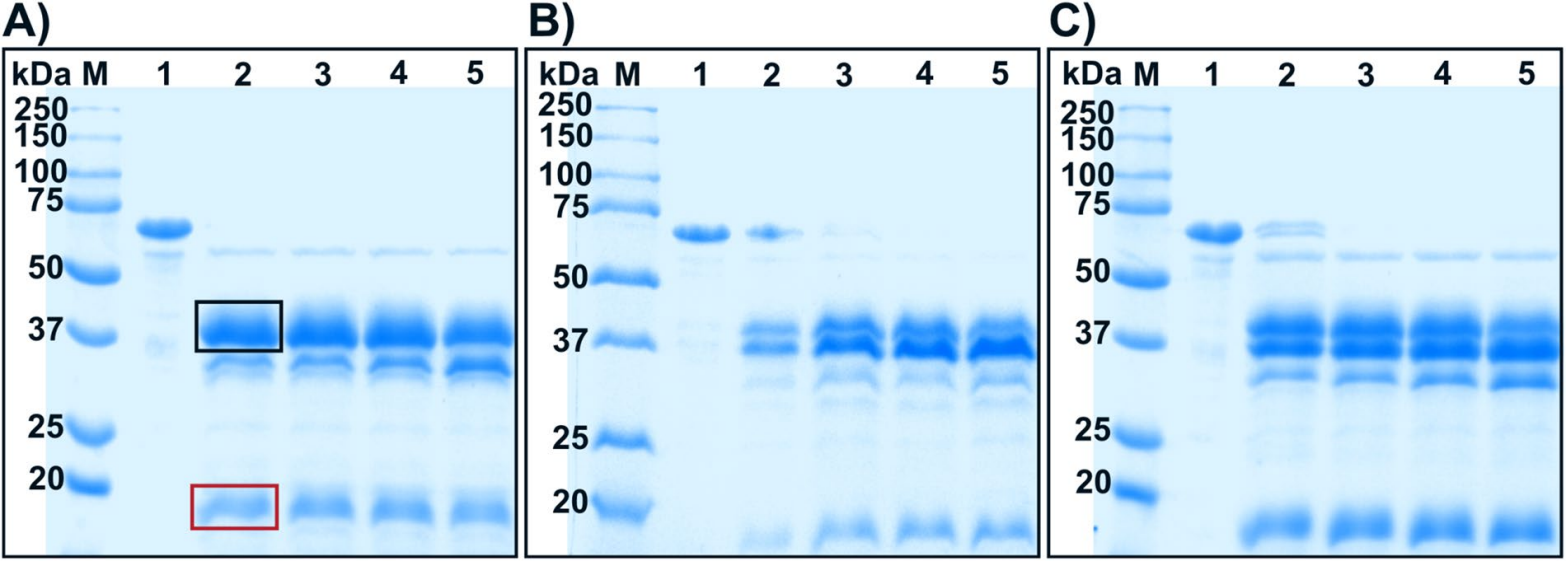
SDS-PAGE gels of pro-*jr*PPO1-wt after digestion with A) trypsin, B) nagarse and C) proteinase K under reducing conditions. Lane M = marker, lane 1 = *jr*PPO1-wt without protease, lanes 2–5 = wild-type with protease (1% w/w) after 1, 2, 3 and 5 minutes. The red box highlights the still intact C-terminal domain and the black box highlights the band representing the active enzyme. Trypsin digestion (**A**) resulted in one sharp band for the active enzyme, thus indicating specific cleavage, whereas nagarse (**B**) and proteinase K (**C**) led to a more diffuse band pattern, indicating cutting at several positions. The gels were cropped to display the lanes of interest. (full-length gels: Fig. S24).

*In vivo* activation of PPOs is achieved by cleavage in-between the C- and N-terminal domain^45^. *In vitro*, PPOs can be activated by, among others, the treatment with proteases, which was attempted for *jr*PPO1-wt using three commercial serine proteases, namely nagarse, trypsin and proteinase K. Proteinase K and nargase prefer cleavage at aromatic and aliphatic amino acid side chains at position P1, while trypsin favors cleavage at a positively charged amino acid like Arg and Lys at position P1. *jr*PPO1-wt was incubated with each protease for different periods of time and the proteolytic reaction was analysed by SDS-PAGE in order to discriminate between specific or non-specific cleavage. The incubation of *jr*PPO1-wt with nagarse and proteinase K resulted in unspecific proteolysis (Fig. 4); however, trypsin cleavage was more specific as latent *jr*PPO1-wt (56 kDa) was cleaved into a 39 kDa peptide representing the active enzyme and a second ∼16 kDa fragment corresponding to the C-terminal domain (Fig. 4).

The activity of *jr*PPO1-wt cleaved by trypsin was tested with the monophenolic substrate tyramine and the enzyme was unexpectedly inactive. Activation of the truncated *jr*PPO1-wt after trypsin cleavage was triggered by SDS (2 mM), resulting in an activity rate similar to the latent *jr*PPO1-wt before trypsin cleavage. Thus, after the treatment with trypsin *jr*PPO1-wt remains in its latent state despite being cleaved. Proteolytic activation of latent PPOs with trypsin has been reported for broad bean PPO^46^, paraguaya peach PPO^47^, spinach PPO^48^, loquat fruit PPO^49^, beet root PPO^19^, grape PPO^50^ and apple PPO^21^; however, when apple PPO (*Md*PPO2) was activated by trypsin, the C-terminal domain was completely digested and only the active domain remained. Hence, cleavage at one precise region of *jr*PPO1-wt, as reported herein, can be insufficient to trigger enzymatic activity. Removing the C-terminal domain by extensive proteolytic cleavage seems to be a prerequisite to convert the enzyme from its latent state into the active form. Otherwise, the still intact C-terminal domain is presumably held in place by non-covalent forces, blocking access to the active center (pre-active form)^16^.

To investigate the proteolytic cleavage of *jr*PPO1-wt by trypsin in detail, the truncated fragment was measured by ESI-MS to identify the cleavage sites (Fig. S3). Two main species of the active domain (39.7 and 39.9 kDa) and one species of the C-terminal domain were detected (16 kDa). In-between those two main domains four and six amino acids were missing, indicating trypsin-cleavage at different positions (Arg348-Val349, Lys350-Lys351 and Lys354-Ala355) (Fig. S4, Table S3). Similarly, cleavage at four conjugated sites (Pro342-Thr343, Thr343-Pro344, Pro344-Arg345 and Arg345-Lys346) was detected *in vivo* for the active domain of *jr*PPO1 purified from leaves^34^. Interestingly, the cleavage sites of trypsin start only two amino acids downstream of the physiological cleavage sites detected in *jr*PPO1 purified from natural sources.

Separation of the two domains after cutting *jr*PPO1-wt with trypsin by ion exchange chromatography (Mono Q 5/50, GE) (Fig. S5) and size-exclusion chromatography (Superdex 200 increase) (Fig. S6) was attempted. However, both methods did not lead to the expected separation of the C-terminal and the active domain. Using the monophenolic substrate tyramine, activity was once again observed only after addition of SDS. Therefore, the interactions in-between the two domains appear to be of significant strength and probably resemble the interactions in-between the active and C-terminal domain of *Md*PPO1 where a self-cleavage reaction in-between those domains did also result in a cleaved but still latent enzyme^16^. Activation of *jr*PPO1-wt was tested using different molarities of salts (NaCl, KCl, MgCl_2_ and CaCl_2_), all of which resulted in active enzyme with the divalent ions (Mg^2+^ and Ca^2+^) requiring lower molarities than the monovalent ions (Na^+^ and K^+^) (Fig. S7).

### Generation, heterologous expression and purification of *jr*PPO1 mutants

In order to investigate the structural basis for mono- and diphenolase activity in *jr*PPO1, five mutants affecting the three amino acid positions Asn240, Leu244 and Phe260 were designed. The mutations were introduced by site-direct mutagenesis (Table S4) and verified by sequencing and ESI-MS. Mutants were expressed and purified as described for *jr*PPO1-wt. The heterologous expression of the five mutants Phe260Gly, Asn240Lys, Leu244Arg, Asn240Lys/Leu244Arg and Asn240Thr/Leu244Arg  yielded adequate amounts of soluble enzyme. The mutants targeted three key amino acid positions of *jr*PPO1: the blocker residue (Phe260) as well as the 1^st^ (Asn240) and 2^nd^ (Leu244) activity controllers. Phe260 has been proposed to stabilize and orient substrates *via π-π*-interactions. The activity controllers are non-conserved amino acids located close to the active center and next to CuB. They have been suspected to control mono- and diphenolase activity. The mutations aimed at the clarification of the role of each of these three amino acid positions in controlling the catalytic activity in *jr*PPO1. Asn240Lys produced the highest yield with 39 mg per liter of culture, while Asn240Lys/Leu244Arg produced the lowest amount of pure enzyme with 11 mg per liter of culture (Table 1). Different yields of recombinant PPO mutants have been described before for c*g*AUS1^51^ and dandelion PPO (*To*PPO-2 and *To*PPO-6)^52^ indicating that mutations influence the expression yield of recombinant PPOs.

**Table 1:** Yields of heterologously expressed *jr*PPO1-wt and the five investigated mutants per liter of culture, copper content *per* enzyme and the molecular weight measured by ESI-MS.

	Yield (mg/l)	Cu-ions/enzyme	Mass (calculated) (Da)	Mass (measured) (Da)	Δ/Da
*jr*PPO1-wt	38	0.8 ± 0.1	56359.4 (−4 H)	56359.5	+0.1
Phe260Gly	39	1.0 ± 0.1	56269.3 (−4 H)	56269.2	−0.1
Asn240Lys	39	0.8 ± 0.1	56373.4 (−4 H)	56373.4	±0
Leu244Arg	22	1.1 ± 0.1	56400.4 (−6 H)	56400.4	±0
Asn240Lys/Leu244Arg	11	1.1 ± 0.1	56414.5 (−6 H)	56414.6	+0.1
Asn240Thr/Leu244Arg	12	0.9 ± 0.1	56387.4 (−6 H)	56387.4	±0

### Molecular mass determination

Molecular masses of recombinant *jr*PPO1-wt and the five mutants were measured by ESI-LTQ-MS. *jr*PPO1-wt features one thioether bridge and two conserved disulfide bonds (Figs. 4 and S8) and exhibits post translation modifications similar to the naturally produced *jr*PPO1^26,34^. The masses of the mutants Phe260Gly and Asn240Lys matched with the calculated masses, including the thioether bridge and one of the two disulfide bonds. In contrast, the masses of Leu244Arg, Asn240Lys/Leu244Arg and Asn240Thr/Leu244Arg revealed the presence of the thioether bridge and both disulfide bonds (Fig. S8 and Table 1). Heterologous expression of *jr*PPO1-wt in *E. coli* entails the possibility of inadequate formation of disulfide bonds since they are unlikely to be formed in the bacterial cytosol due to the reducing environment^53^. However, the disulfide bonds can be formed in the mass spectra as a result of the electro spray ionization process (one-electron oxidation of thiol groups leads to the formation of thiyl radicals which rapidly dimerize)^54^. On the other hand, the thioether bridge is believed to be formed independently by the active copper center *via* an autocatalytic process and is thus likely to be present^55^.

### Copper content determination

Copper content was determined for *jr*PPO1-wt and the five investigated mutants spectrophotometrically by measuring the absorption (546 nm, ε = 6300 M^−1^ cm^−1^) of a Cu^I^-2, 2′-biquinoline complex. *jr*PPO1-wt contains 0.8 ± 0.01 copper atoms per active site and all the mutants showed similar values of ~ one copper per protein (Table 1). Therefore, the copper content does not account for the different activities of the investigated mutants.

**Figure 5:**
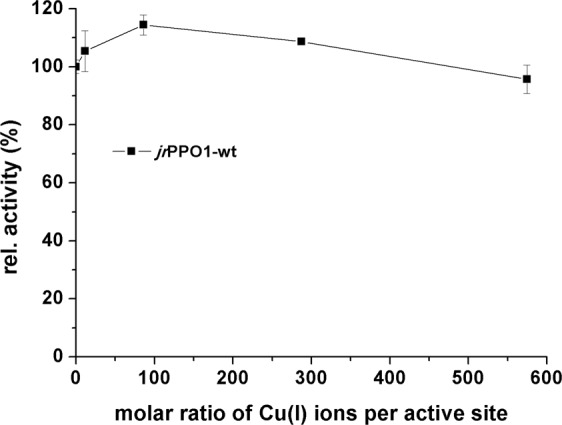
Influence of Cu^I^ ions on *jr*PPO1-wt activity. CuCl was added to the latent *jr*PPO1-wt (0.17 µM) in a molar ratio shown by the x-axis. The y-axis shows the change of the specific activity in relation to the enzyme without additional copper.

*jr*PPO1-wt was incubated with varying molarities of copper ions in a molar ratio from 1:10 to 1:575 to test its ability to assimilate copper ions post heterologous expression. Specific activity with 1 mM tyramine increased up to a maximum of 114% in comparison with the enzymatic activity in the absence of additional copper (Fig. 5). Thus, in PPOs, the copper co-factor can only be partially incorporated into the active site after the expression and the folding process.

### Secondary structure investigation by CD-spectroscopy

**Figure 6:**
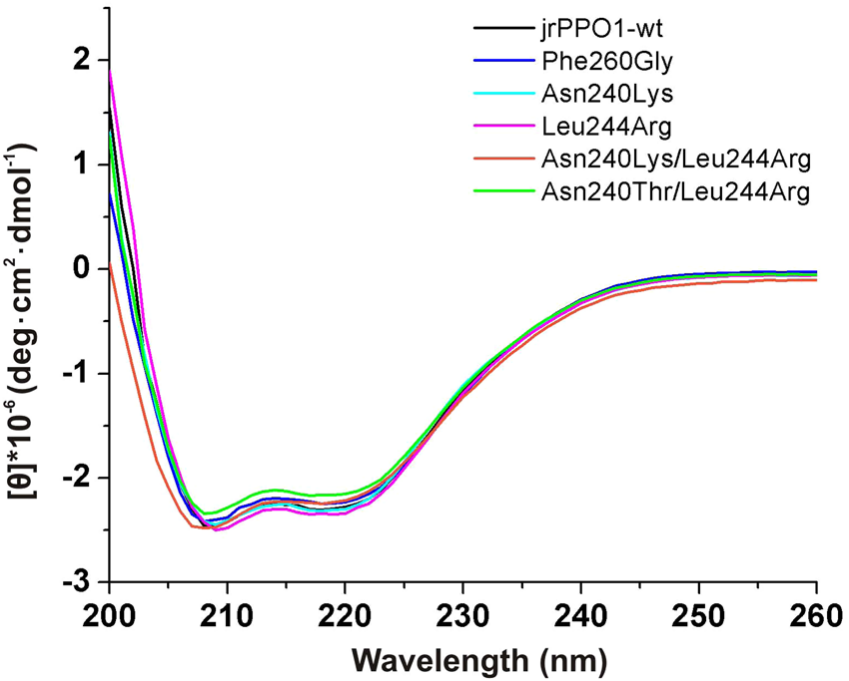
CD-spectra of *jr*PPO1-wt and the five kinetically investigated mutants. Samples were prepared at a protein concentration of 1 mg/ml in a buffer containing 50 mM sodium phosphate (pH 7.8) and 150 mM NaCl. Spectra are presented after buffer subtraction.

CD (circular dichroism) spectroscopic studies were performed to evaluate the influence of the mutations on secondary structure elements and correct folding. The CD-spectra of *jr*PPO1-wt and the five mutants show prominent bands at 208  and 222 nm (Fig. 6), corresponding to alpha helices. The high similarity of the spectra indicates no change in alpha helicity. Thus, the mutations do not influence protein folding and secondary structure formation.

### Kinetic characterization of recombinant *jr*PPO1-wt

Recombinant *jr*PPO1-wt was characterized in terms of pH and SDS dependence using tyramine and dopamine (see supporting information, Fig. S9). The highest activities were measured for both substrates at pH 6.0 and 2 mM SDS (Fig. S10). Thus, the substrate does not influence the pH and SDS optima of *jr*PPO1-wt. Kinetic measurements were performed under these conditions by measuring the increase of the colored reaction products photometrically (Fig. S11). Substrate specificity of the recombinant latent *jr*PPO1-wt was characterized kinetically with the monophenolic substrates *L*-tyrosine and tyramine as well as the diphenolic substrates dopamine and *L*-DOPA (Table 2). Recombinant *jr*PPO1-wt shows higher affinity and activity towards the monophenolic substrate tyramine (*k*_cat_ = 24.7 s^−1^ and *K*_m_ = 0.451 mM) in comparison to *L*-tyrosine (*k*_cat_ = 6.0 s^−1^ and *K*_m_ = 1.42 mM, Table 2). Regarding diphenolase activity, recombinant *jr*PPO1-wt exhibits higher specificity for dopamine (*K*_m_ = 0.75 mM) than for *L*-DOPA (*K*_m_ = 6.2 mM); however, kinetic data indicate a higher activity rate of *jr*PPO1-wt with *L*-DOPA (*k*_cat_ = 111.2 s^−1^) than with dopamine (*k*_cat_ = 92.5 s^−1^).

**Table 2:** Kinetic parameters for recombinant *jr*PPO1-wt, the five investigated mutants and *jr*PPO1 purified from natural sources (previously published) with the monophenolic substrates tyramine and *L*-tyrosine and the diphenolic substrates dopamine and *L*-DOPA.

Enzyme/Mutant	tyramine			*L*-tyrosine		
	*k*_cat_ (s^−1^)	*K*_m_ (mM)	*k*_cat_/*K*_m_ (s^−1^ mM^−1^)	*k*_cat_ (s^−1^)	*K*_m_ (mM)	*k*_cat_/*K*_m_ (s^−1^ mM^−1^)
*jr*PPO1-wt (recombinant)	**24.7** **±** 1.5	**0.451** **±** 0.084	**55** **±** 11	**6.0** **±** 1.1	**1.42** **±** 0.37	**4.3** **±** 1.4
Phe260Gly	**0.323** **±** 0.025	**3.05** **±** 0.72	**0.103** **±** 0.026	n.a.*	n.a.*	**0.00783** **±** 0.00049
Asn240Lys	**0.0542** **±** 0.0034	**8.2** **±** 1.4	**0.0066** **±** 0.0012	n.a.*	n.a.*	
Leu244Arg	**1.62** **±** 0.09	**2.68** **±** 0.53	**0.60** **±** 0.12	n.a.*	n.a.*	**0.624** **±** 0.0068
Asn240Lys/Leu244Arg	**n.a**.	**n.a**.		**n.a**.	**n.a**.	
Asn240Thr/Leu244Arg	**n.a**.	**n.a**.		**n.a**.	**n.a**.	
*jr*PPO1 (natural source)	**18.3**^26^	**0.27**^26^		**2.7**^26^**–20.80**^34^	**1.02**^26^**–1.90**^34^	
**Enzyme/Mutant**	**dopamine**			***L*-DOPA**		
*jr*PPO1-wt (recombinant)	**92.5** **±** 7.8	**0.75** **±** 0.13	**123** **±** 24	**111.2** **±** 8.8	**6.2** **±** 1.0	**18.1** **±** 3.4
Phe260Gly	**7.36** **±** 0.98	**1.2** **±** 0.4	**5.9** **±** 2.2	**9.1** **±** 1.6	**10.5** **±** 3.4	**0.86** **±** 0.32
Asn240Lys	**2.51** **±** 0.40	**19.8** **±** 4.7	**0.132** **±** 0.097	**1.08** **±** 0.15	**16.7** **±** 3.7	**0.065** **±** 0.017
Leu244Arg	**24.9** **±** 1.6	**2.36** **±** 0.41	**10.6** **±** 2.0	**10.5** **±** 1.2	**4.2** **±** 1.2	**2.51** **±** 0.78
Asn240Lys/Leu244Arg	**0.029** **±** 0.002	**6.8** **±** 1.3	**0.00428** **±** 0.00085	**0.104** **±** 0.019	**16.5** **±** 4.8	**0.0060** **±** 0.0021
Asn240Thr/Leu244Arg	**0.789** **±** 0.079	**6.6** **±** 1.5	**0.122** **±** 0.026	**0.180** **±** 0.027	**13.1** **±** 3.3	**0.0129** **±** 0.0040
*jr*PPO1 (natural source)				**199.3**^34^	**8.80**^34^	

n.a. indicates substrate-enzyme combinations that showed no activity. n.a.* represents samples that were active but could not be measured due to extensively reduced reactivity and increased *K*_m_ values in combination with limited substrate solubility. Measurements were performed in triplicates. The numbers represent mean values ± one standard deviation.

The kinetic behavior of heterologously expressed *jr*PPO1-wt was compared with the enzyme isolated from its natural source^26,34^. *K*_m_ and *k*_cat_ values were determined for the investigated monophenolic (tyramine and *L*-tyrosine) and diphenolic substrates (dopamine and *L*-DOPA). These values are within the range of previously published studies for the enzyme purified from natural sources (Table 2) and, therefore, demonstrate that the heterologous expression system of *E. coli* does not affect the activity of the enzyme.

### Substrate scope assays of *jr*PPO1-wt and the five investigated mutants

**Figure 7:**
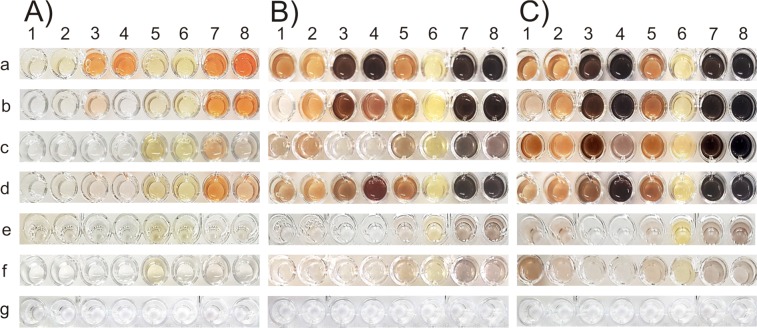
Substrate scope assay of *jr*PPO1-wt and the investigated mutants. Eight substrates were tested after different periods of time: A) 5 minutes, B) 3 hours and C) 20 hours. 100 µg of latent *jr*PPO1 were mixed with 1 mM substrate in a 50 mM phosphate buffered solution (6.0 pH) containing 2 mM SDS as an activator in a total volume of 200 µl. a = *jr*PPO1-wt, b = Phe260Gly, c = Asn240Lys, d = Leu244Arg, e = Asn240Lys/Leu244Arg, f = Asn240Thr/Leu244Arg, g = control assay without enzyme. 1 = phenol, 2 = tyrosol, 3 = tyramine, 4 = *L*-tyrosine, 5 = catechol, 6 = 4-tert-butylcatechol, 7 = dopamine and 8 = *L*-DOPA.

Substrate scope assays were performed using four monophenols (phenol, tyrosol, tyramine and *L*-tyrosine) and four diphenols (catechol, 4-tert-butylcatechol (4-TBC), dopamine and *L*-DOPA) (Figs. S9 and S12-S16). The measurements revealed the preference of *jr*PPO1-wt towards certain substrate characteristics, such as polarity, charge and steric properties (Fig. 7). *jr*PPO1-wt displayed high activity towards all tested substrates with higher reaction rates towards diphenols compared to their corresponding monophenols (Fig. 7).

The first mutant Phe260Gly accepted all substrates used in the substrate scope experiment, exhibiting the lowest activity with phenol (Fig. 7). Arguably, this demonstrates that most substrates (tyramine, *L*-tyrosine and tyrosol, Fig. S9) are able to partially compensate the loss of *π-π*-interactions probably by interacting with  other side chain residues.

Asn240 at the position of the 1^st^ activity controller was previously reported to discriminate between monophenolase and diphenolase activity^56^. However, numerous tyrosinases feature a different amino acid at this position, such as *Md*PPO1 (Ala239) and *Md*PPO3 (Gly239)^21^, larreatricin hydroxylase (*Lt*PPO) from *Larrea tridentata* (Gly241)^57^ and *Sl*PPO1 (Ser242)^58^ from *Solanum lycopersicum*. Asn240 stabilizes cooperatively with a conserved glutamic acid (Glu235) a conserved water molecule *via* hydrogen bonding. The water molecule activated by Glu235 acts as a primary proton acceptor by abstracting a proton from the hydroxyl group of an incoming phenolic substrate, which is subsequently transferred  to the carboxylic group of the conserved glutamic acid^59^. However, the replacement of Asn240 at the 1^st^ activity controller position cannot transform a TYR into a CO since Asn240Lys was still active on the monophenolic substrates phenol, tyrosol, tyramine and tyrosine.

Similarly, the mutant Leu244Arg was found to accept all four investigated monophenols and does, therefore, not discriminate between mono- and diphenolase activity.

To eliminate monophenolase activity, double mutations targeting the 1^st^ and 2^nd^ activity controllers were designed (Asn240Lys/Leu244Arg and Asn240Thr/Leu244Arg). Asn240Lys/Leu244Arg did not accept standard tyrosinase substrates (tyramine and *L*-tyrosine) (Fig. 7); however, phenol and tyrosol were still accepted (after a reaction time of approximately 20 hours) as demonstrated by the substrate scope assay (Fig. 7). On the other hand, the mutant was active on all tested diphenolic substrates (Fig. 7). Therefore, the mutation Asn240Lys/Leu244Arg converts *jr*PPO1 into a CO with specific monophenolase activity. Furthermore, the double mutant Asn240Thr/Leu244Arg was designed targeting the two activity controllers, which resembles the activity controller residues of *cg*AUS1(Thr253 and Arg257)^25^. According to the substrate scope assay, the mutant was also classified as a CO since it did no longer accept tyramine and *L*-tyrosine (Fig. 7).

### Kinetic investigation of *jr*PPO1 mutants

Kinetic assays were performed to further investigate and quantify the influence of the mutations. Mutating Phe260 to glycine (Phe260Gly) impaired both activities (monophenolase and diphenolase). The *k*_cat_ values of tyramine, dopamine and *L*-DOPA were reduced 77-fold (*k*_cat_ = 0.323 s^−1^), 13-fold (*k*_cat_ = 7.36 s^−1^) and 12-fold (*k*_cat_ = 9.1 s^−1^), respectively (Table 2). Therefore, our data verify that the *π-π*-interactions between the phenolic ring of an incoming substrate and the aromatic ring of the gatekeeper residue (Phe260) and the 2^nd^ histidine of CuB can stabilize the correct orientation of the substrate.

For Asn240, the *k*_cat_ value for tyramine was reduced 457-fold, while the *k*_cat_ values for the diphenols dopamine and *L*-DOPA were reduced 37-fold and 103-fold, respectively, in comparison to *jr*PPO1-wt (Table 2). The ratio (*K*_m_/*k*_cat_)_tyramine_/(*K*_m_/*k*_cat_)_dopamine_, which expresses the preference of an enzyme for mono- or diphenols, was reduced 8.8-fold (0.45 for *jr*PPO1-wt versus 0.051 for Asn240Lys). Thus, the mutant Asn240Lys demonstrates that the amino acid at the 1^st^ activity controller position can influence the tyrosinase activity but is not able to completely shut down monophenolase activity.

Leu244 at the 2^nd^ activity controller position of *jr*PPO1-wt was mutated to a positively charged arginine (Leu244Arg), leading to a decrease of the *k*_cat_ values for mono- and diphenolic substrates by 15-fold for tyramine, 4-fold for dopamine and 11-fold for *L*-DOPA in comparison to *jr*PPO1-wt (Table 2). However, the affinity towards the diphenolic substrate *L*-DOPA, which features a carboxyl group, increased (*K*_m_ = 4.2 mM) in contrast to dopamine, which showed a decreased affinity (*K*_m_ = 2.36 mM, Table 2). Thus, the data suggest that strong interactions between the 2^nd^ activity controller and the aliphatic chain attached to the phenolic group of a substrate increase the specificity of the enzyme towards its substrates (tyramine, tyrosine dopamine, L-DOPA). This trend, however, was not found to correlate with the turn-over rate of the mutant (Table 2). The role of this amino acid in substrate selection seems to be a general characteristic of PPOs since similar results were reported for *To*PPO-2 and *To*PPO-6. The reaction between those enzymes and 3,4-dihydroxyphenylacetic acid, which displays a carboxylic group in its side chain (like *L*-DOPA), was associated with lower *K*_m_ values when an arginine was present at this position (*To*PPO-6) rather than an isoleucine (*To*PPO-2)^52^. Therefore, PPOs featuring a hydrophobic amino acid at the 2^nd^ activity controller position do most likely target substrates with a less polar aliphatic chain, whereas substrates with a negatively charged group at their aliphatic chain are more readily accepted by PPOs carrying an arginine at this position. It can be concluded that the 2^nd^ activity controller influences both diphenolase and tyrosinase activity and acts as a substrate selector, but it is not able to inhibit monophenolase activity.

For the double mutations, both the affinity and the turn-over rate were impaired as the *K*_m_ valuefor dopamine increased 9-fold and that for *L*-DOPA 3-fold compared to the wild type. , The *k*_cat_ values decreased also significantly, by 3190-fold in the case of dopamine and by 1112-fold in that of  *L*-DOPA (Table 2). Asn240Thr/Leu244Arg was designed to increase diphenolase activity while suppressing monophenolase activity. This double mutant was substantially more active on diphenols than Asn240Lys/Leu244Arg, which was demonstrated by the *k*_cat_ value for dopamine, which was 27-fold increased.

Sequence alignments of biochemically characterized plant PPOs show that COs exclusively feature Thr as the 1^st^ activity controller and in most cases display Arg as the 2^nd^ activity controller (except for *Sl*PPO2, which displays Ile as the 2^nd^ activity controller), whereas TYRs possess a vast variety of amino acids at the 1^st^ and 2^nd^ activity controller position (Fig. S1). However, the sequence of *To*PPO-5 (TYR)^60^ and mutants of dandelion PPO (*To*PPO-2)^32^ prove that TYRs can still function on monophenols, even though Thr is present at the 1^st^ activity controller position. Kinetic data of the herein presented *jr*PPO1 mutant Leu244Arg demonstrate that Arg at the 2^nd^ activity controller position does not convert a TYR into a CO either. However, the double mutant Asn240Thr/Leu244Arg clearly shows that Thr as the 1^st^ activity controller in combination with Arg as the 2^nd^ activity controller converts the TYR from walnut (*jr*PPO1) into a CO as it did no longer accept tyrosine and tyramine. Moreover, this double mutant demonstrates that different amino acids can be present at the 1^st^ activity controller position of COs. In accordance with our kinetic data (Table 2), it can be assumed that Thr at this position is associated with increased catalytic activity.

Based on the residual monophenolase activity towards phenol and tyrosol in Asn240Lys/Leu244Arg and Asn240Thr/Leu244Arg, we propose that PPOs may show activity towards non-standard monophenolic substrates (secondary metabolites of *Juglans regia*) despite not accepting tyrosine or tyramine.

### Molecular docking

Molecular Docking was performed with *jr*PPO1-wt and all reported mutants using tyramine, *L*-tyrosine, dopamine and *L*-DOPA as substrates. The calculated binding poses offer highly valuable information and provide insights into the possible effects of each mutation on the enzymatic activity (Table S5).

Docking studies with *jr*PPO1-wt revealed that the main driving force for the correct substrate orientation is the *π*-stacking system established between the aromatic ring of the gatekeeper residue Phe260, the substrate’s aromatic ring and the imidazole group of the CuB coordinating His243 (*π*-stacking system: Phe260-substrate-His243) (Fig. S17). According to literature, Asn240 is involved in the stabilization of a conserved water molecule, which is required for the deprotonation of monophenolic substrates; however, the docking results indicate that Asn240 also interacts with the tail of all substrates, providing additional stabilization of the substrate’s orientation and binding position. Moreover, the more hydrophobic substrates tyramine and dopamine could be further stabilized by the hydrophobic 2^nd^ activity controller Leu244, which is supported by the experimental results (*K*_m_ values: tyramine = 0.451, dopamine = 0.75 versus tyrosine = 1.42 and *L*-DOPA = 6.2).

In Phe260Gly, the *π*-stacking system is destructed by the loss of the aromatic Phe260, therefore, incoming substrates are no longer sufficiently stabilized. However, to some extent, reasonable binding is still possible as long as the substrate is stabilized by His243 (*via π*-interaction of the aromatic ring) and the 1^st^ activity controller Asn240 (Fig. S18).

Docking substrates into the active site of the mutant Asn240Lys suffered from steric and entropic problems as Lys at position 240 oscillates (as evidenced by docking showing a wide range of different Lys240 positions), thereby obstructing access to the active site. Due to its high flexibility and positive charge, Lys240 additionally disrupts potential H-bond-networks, which are required for the catalytic reaction of the enzyme. Lys240 is positioned close to Glu235 (~2.0 Å), which together with Asn240 stabilizes a conserved water molecule. Therefore, Lys240 can exhibit strong ionic interactions with the negatively charged Glu235, thus considerably weakening or even completely inhibiting its water-stabilizing function (Fig. S19). Furthermore, the position of Lys240 in the best docking pose overlaps with the position of the conserved water molecule. If the deprotonation of monophenolic substrates takes still place, Lys240 could directly interact with the emerging phenolate-intermediate and thus interfere with the following steps of the monophenolase activity. However, since the Phe260-substrate-His243 *π*-stacking system can still be formed, AutoDock produced binding poses with ‘reasonable’ orientation for all the substrates within the active site (Fig. S19).

Similar to Asn240Lys, Leu244Arg also suffered from steric and entropic issues. Arginine at position 244 does not interfere with Glu235 and the substrate-deprotonation-process, as indicated by significantly lower *K*_m_ values for Leu244Arg compared to Asn240Lys (Table 2). The docking results suggest that the positively charged guanidinium group of Arg240 interacts strongly with the carboxylate group of *L*-tyrosine and *L*-DOPA, whereas the amine group (which is predominantly positively charged at the investigated pH) of the other two substrates is electrostatically repelled (Fig. S20). Presumably, the interaction between Arg240 and the carboxylate group of *L*-DOPA and *L*-tyrosine (which are most probably of ionic nature as the amide group is mainly charged at the investigated pH) is too strong, resulting in the amide-containing substrates (tyramine and dopamine) being held in place by Arg240. Thus, these substrates are unable to reach the active center. This could explain the higher activity of this mutant with tyramine and dopamine. However, considering the above described scenarios, it is still unclear why *L*-DOPA has a higher affinity towards Leu244Arg (*K*_m_ = 4.2 mM) than towards *jr*PPO1-wt (*K*_m_ = 6.2 mM).

For Asn240Lys/Leu244Arg, the best docking poses suggest that all substrates can be orientated correctly within the active site since it exhibits the Phe260-substrate-His243 *π*-stacking-system (Fig. S21). Nevertheless, significantly reduced activity was observed for all tested substrates. As shown by the docking poses, the positively charged Arg244 and Lys240 will electrostatically avoid each other and, therefore, might not be able to adopt the position required to exhibit the same effect as in the case of the respective single mutant (e.g. Lys240 will not be able to interact with Glu235 because it is repelled by Arg244 and will constantly change its position). Thus, it is suggested that the affinity-changing effects of both Arg244 and Lys240 are mutually weakening instead of mutually reinforcing. On the other hand, the simultaneous presence of both Arg244 and Lys240 leads to a less readily accessible active site, especially when considering the flexible nature of both residues, which leads to the shape of the active site pocket changing with the oscillation frequency of Arg244 and Lys240. Thus, the substrates might struggle to adopt the correct orientation for the deprotonation or oxidation step, which could be the main reason for the decreased activity of this double mutant. The fact that the diphenolic substrates are able to cope better with the double mutation than the monophenolic substrates further indicates interference with the deprotonation step as it was proposed that diphenols do not require prior deprotonation^61^.

The docking as well as the kinetic results of Asn240Thr/Leu244Arg are similar to those of Asn240Lys/Leu244Arg (Fig. S22). Due to the mutation of Asn240 to Thr240, the substrate-stabilizing contribution of the 1^st^ activity controller is lost. Regarding the chemical features, threonine should in principle be able to stabilize the conserved water molecule *via* hydrogen bonds and thus ensure the deprotonation process. However, the low activity of this mutant suggests that larger disturbances are caused by the introduction of Thr.

The amide group of asparagine can form up to four hydrogen bonds, two donated by the nitrogen and two accepted by the oxygen atom. In contrast to glutamine, the side chain of asparagine is located closer to the main chain and thus tends to form side chain - main chain hydrogen bonds. Considering this feature, Asn240 is able to form hydrogen bonds with the conserved water molecule, the copper-coordinating His239, the backbone carbonyl group of Glu235 and the substrate (if it contains a functional group that is able to form H-bonds at its tail). There are some oxidoreductases, whose activity depends on proximal histidine-asparagine interactions (e.g. mammalian peroxidases), where the asparagine acts as an H-acceptor ensuring that the catalytic histidine is present in its imidazolate form (deprotonated histidine)^62^. In this way, the asparagine residue creates a negative redox potential within the active site, which is required for the catalytic reaction, as the imidazolate strengthens the metal-histidine bond, which in turn stabilizes the metal in its higher/highest oxidation state. This could also hold true for PPOs, explaining the importance of Asn240 as the 1^st^ activity controller for the activity of most PPOs. Furthermore, Glu235 is located on a small alpha-helical unit which is preceded by a small loop and, therefore, the position of Glu235 might not be sufficiently stable; however, Asn240 is hydrogen bonded to the backbone carbonyl of Glu235 stabilizing its position. This hydrogen bond is only possible due to the proximity of the amide group of Asn240 to the backbone (e.g. glutamine would be too long to exhibit the same hydrogen bond). Therefore, it is suggested that the mutation of Asp240 (into Thr240 and Lys240) leads to the disturbance of the above described hydrogen-network, which ultimately affects the enzyme’s activity.

## Conclusions

In this study, *jr*PPO1 from *Juglans regia* was, for the first time, heterologously expressed in *E. coli*, purified and characterized kinetically and biochemically in its latent form. Latent *jr*PPO1-wt shares a high similarity in its activity with the isoenzyme purified from natural source^26,34^ and demonstrates that heterologous expression of *jr*PPO1-wt does not affect biochemical characteristics. Trypsin activation converted the latent *jr*PPO1-wt to a pre-active form as the cleavage sites (Arg348-Val349, Lys350-Lys351 and Lys354-Ala355) are located within the linker region (Pro336-Pro375, Fig. S4), which connects the active with the C-terminal domain. *jr*PPO1-wt truncated by trypsin exhibits characteristics similar to latent *jr*PPO1-wt and its activation was induced only after treatment with SDS. Mutations applied to the key residues Phe260 (gatekeeper), Asn240 (1^st^ activity controller) and Leu244 (2^nd^ activity controller) led to significant differences in the substrate preference of the enzyme. The mutant Phe260Gly proved the importance of the bulky phenylalanine residue at the gatekeeper position of plant PPOs. Monophenolase and diphenolase activity of Phe260Gly were impaired, demonstrating that the described *π-π*-interactions between the gatekeeper residue and the phenolic ring of the putative substrate are obligatory for the correct performance of PPOs. Single mutations (Asn240Lys and Leu244Arg) affecting the 1^st^ and 2^nd^ activity controller residues failed to inhibit the tyrosinase activity of *jr*PPO1-wt completely. Although monophenolase activity was strongly impaired, the mutants still exhibited weak activity with the classical monophenolic substrates (tyramine and/or *L*-tyrosine). However, complete inactivation of monophenolase activity in *jr*PPO1-wt was achieved only when both activity controller residues were mutated (Asn240Lys/Leu244Arg and Asn240Thr/Leu244Arg). The two double mutants were completely inactive on the standard substrates tyramine and *L*-tyrosine, exhibiting only weak monophenolase activity towards phenol and tyrosol. Molecular docking confirmed the kinetic data, suggesting that the *π-π*-stacking system as well as non-covalent interactions between the 1^st^ and 2^nd^ activity controller and the tail of substrates are responsible for substrate stabilization and correct orientation. Furthermore, Asn240 is suggested to critically influence monophenolase activity by creating a negative redox potential within the active site. Based on the copper content measurements, all recombinantly produced proteins (*jr*PPO1-wt and five mutants) contain a similar amount of copper (~0.8–1.1 copper ions per protein), indicating that the changes in activity are not based on the number of copper ions in the active center. The investigation of the amino acid residues controlling the two activities (monophenolase and diphenolase) in PPOs and the quantification of their influence on the catalytic activity represent a tough challenge in this field and has remained unclear over the last decades. This study provides valuable insights into which amino acid positions should be manipulated for the complete inhibition of monophenolase activity in PPOs.

## Materials and Methods

### Cloning of *jr*PPO1-wt and generation of the mutations

Walnut leaves were collected from trees around Vienna and stored at −80 °C. Total RNA was isolated from 500 mg of frozen leaves using the *RNeasy Plant Mini Kit* (Qiagen, Hilden, Germany). Subsequently, cDNA was synthesized using a poly-T Primer and the gene coding the latent *jr*PPO1-wt was amplified using specific primers (Table S4) and *Q5*^*®*^ *High-Fidelity DNA polymerase* (NEB, Ipswich, USA). The gene was cloned into the pENTRY-IBA51 vector, sequenced and the construct was further subcloned into the open reading frame of a pGEX-6P-1 based expression vector using the *Esp3I* restriction enzyme (Thermo Fisher, Waltham, USA). In addition, the vector carrying the *jr*PPO1-wt gene was used as a template for the mutagenesis experiments. Mutations were introduced into the *jr*PPO1-wt gene by back to back annealing primers with the forward primer carrying the mutation. An amplicon was obtained using *Q5*^*®*^ *High-Fidelity DNA Polymerase* (NEB). T4 Polynucleotide Kinase (NEB) phosphorylated DNA ends and *T4 DNA Ligase* (NEB) created cyclic plasmids (pENTRY-IBA51). The open reading frames of the mutants were then subcloned into pGEX-6P-1 using the *Esp3I* restriction enzyme. The expression vector pGEX-6P-1 carried an N-terminal glutathione-S-transferase tag (GST-tag) with a recognition site for the human rhinovirus 3C protease (HRV-3C) between the tag and the target gene and was used for the expression of *jr*PPO1-wt after transforming it into chemically competent *E. coli* BL21 (DE3) cells.

### Heterologous expression and purification

The expression of the fusion protein under the control of a tac promoter was performed in a 2xYT-medium (1.6% tryptone, 1% yeast extract, 0.5% NaCl) containing ampicillin (100 µg/ml). An overnight culture was grown in LB-medium (1% tryptone, 1% NaCl, 0.5% yeast extract) from a freshly transformed single colony and was used to inoculate the expression batches. The culture was incubated at 37 °C and 240 rpm until the OD_600_ reaches a value of 1.5. Afterwards, it was cooled at 20 °C and supplemented with 0.5 mM CuSO_4_ and 0.5 mM isopropyl β-D-1-thiogalactopyranoside (IPTG). The cultures were shaken at 20 °C and 240 rpm for 65 hours. After incubation, the batches were centrifuged at 4000 × g for 25 minutes and the pellets were re-suspended in lysis buffer (200 mM NaCl, 50 mM Tris-HCl, 5 mM EDTA, 2 mM benzamidine, 1 mM phenylmethylsulfonylfluoride and 0.5 g/l lysozyme, pH 7.8). The solution was frozen in liquid nitrogen, followed by thawing in a 15 °C water bath repetitively three times. Subsequently, 0.03 g/l DNase I and 7 mM MgCl_2_ were added in order to hydrolyse deoxyribonucleic acids and reduce the viscosity of the lysate. The solution was incubated for 15 minutes on ice and subsequently centrifuged at 30000 × g for 45 minutes and filtered before being applied to a 5 ml GSTrap FF column (GE Healthcare, Freiburg, Germany) *via* an ÄKTA explorer system placed in a refrigerator at 4 °C. The GST-tagged protein was trapped on the column and purged using a running buffer (200 mM NaCl and 50 mM Tris-HCl at pH 7.8) before being eluted with 200 mM NaCl, 50 mM Tris-HCl and 10 mM reduced glutathione at pH 7.8. The purified GST-*jr*PPO1-wt fusion protein was exchanged to a glutathione-free buffer containing 200 mM NaCl and 50 mM Tris-HCl at pH 7.8 and 1 mM dithiothreitol (DTT) using a Vivaspin ultrafiltration device (VWR, molecular weight cut-off 30 kDa). A GST-tagged HRV-3C-protease produced in house^39^ was added in a mass ratio of 1: 50 (protease: protein) and the mixture was placed at 4 °C for 40 hours. The solution was then again applied to a 5 ml GSTrap FF column, separating the GST-tag and the GST-protease from *jr*PPO1-wt, which passed through the column and was collected. Quantification of the protein was performed using the Lambert-Beer law, the absorption of the protein was measured at 280 nm and extinction coefficients were calculated by the ExPASy ProtParam tool^63^. The purity of the final protein was checked *via* SDS-PAGE gel under denaturing conditions.

### Molecular mass determination *via* mass spectroscopy

Mass spectra were obtained using LTQ Orbitrap Velos mass spectrometer (Thermo Fisher Scientific, Bremen, Germany) equipped with a nanospray ion source (electrospray voltage: 2.1 kV, ion transfer capillary temperature: 300 °C), coupled to a nano HPLC-system (UltiMate 3000, Dionex). 5 µl of the sample was first loaded on a trap column with 0.1% trifluoroacetic acid. Separation was carried out on a C4 analytical column 50 cm × 75 µm Accucore C4, 2.6 µm, 150 Å (Thermo Fisher Scientific) at a flow rate of 300 nl/min. Mobile phase A: 2% acetonitrile, 98% H_2_O, 0.1% formic acid. Mobile phase B: 80% acetonitrile, 20% H_2_O, 0.1% formic acid. Full MS scans were acquired in positive ion mode at 400–2000 m/z range at a resolution of 7500 (FWHM at 400 m/z).

### Copper content determination

The copper content was determined for the investigated enzyme and the five mutants according to a method published by Hanna *et al*.^64^. In short, 600 µg purified latent enzyme were mixed with 50 mM sodium ascorbate, diluted to a total volume of 400 µl with 100 mM sodium phosphate buffer at pH 6.0 and 600 µl of a 0.5 g/l 2,2′-biquinolinein glacial acetic acid solution were added. The formation of a copper-2,2′-biquinoline complex was measured at 546 nm (ε = 6300 M^−1^ cm^−1^) after incubating for ten minutes and copper contents were calculated for blank corrected samples. Measurements were applied in triplicates.

### CD-spectra of *jr*PPO1-wt, the five mutants investigated kinetically

Spectra were recorded on a Chirascan Plus spectropolarimeter (Applied Photophysics) purged sufficiently with N_2_ before use. All the samples were prepared at a protein concentration of 1 mg/ml in a buffer containing 50 mM sodium phosphate (pH 7.8) and 150 mM NaCl. Protein concentrations were determined spectrophotometrically. CD spectra were recorded at 20 °C. The measurements were recorded from 200 to 260 nm using a precision quartz cuvette with a path length of 1 mm and the spectra obtained represent the average of five scans at a scan rate of 60 nm/min. The final spectra are presented after buffer subtraction.

### Proteolytic activation of *jr*PPO1-wt

Proteolytic activation of latent *jr*PPO1-wt was investigated using three different serine proteases, namely nagarse (subtilisin BPN, *Bacillus amyloliquefaciens*), trypsin and proteinase K. They were mixed with *jr*PPO1-wt in a mass ratio of 1:100 in 300 mM Tris-HCl at pH 7.8. 100 mM Na-ascorbate was added as a reductant to suppress tyrosinase side reactions and proteolysis was carried out for different periods of time before being stopped by the addition of 10 mM PMSF. Proteolytic digestion was checked by reducing SDS-PAGE gel. Purification of the digested samples was carried out *via* an ion exchange chromatography (Mono Q 5/50 GL column GE) with the running buffer: 10 mM Tris-HCl pH 7.8 and a size exclusion chromatography on a Superdex 200 increase column (GE) using a running buffer: 200 mM NaCl, 50 mM Tris-HCl, pH 7.8.

### Investigation of pH optimum and activation by SDS

Spectroscopic measurements were all carried out on TECAN infinity M200 (Tecan, Salzburg, Austria) in 96 well plates. The latent enzyme was used for investigating the pH-dependence and activation by SDS. The activity was determined using the substrates tyramine and dopamine (1 mM) and by measuring the accumulation of the colored reaction product spectrophotometrically at 400 nm and 25 °C with 2.5 mM SDS. Different pH values ranging from pH 3.0 to 8.0 in steps of 0.5 pH units (pH 3 to 5.5 = sodium citrate buffer, pH 6 to 8 = phosphate buffer; ionic strength of the buffers used is indicated in Table S6) were investigated showing its maximum activity at pH 6.0 in a 50 mM phosphate-buffered solution. Moreover, to overcome the obstacle of its latency, the enzyme was activated with different SDS concentrations which resulted in highest levels of activity at 2 mM SDS. Hence, the influence of the pH on the activity was reassessed using 2 mM SDS, which once again led to an optimum at pH 6.0. Subsequently, kinetic measurements were carried out at pH 6.0 using 2 mM SDS as an activator. Furthermore, the pH and SDS optima of *jr*PPO1-wt were determined using 1 mM dopamine and *L*-DOPA as substrates.

### Kinetics and substrate scope assays of *jr*PPO1-wt and mutants

Activities were determined spectrophotometrically for two monophenolic (tyramine and *L*-tyrosine) and two diphenolic substrates (dopamine and *L*-DOPA). By detecting the appearance of the colored quinones on a TECAN infinite M200 absorption curves were recorded at different substrate molarities in a total volume of 200 µl containing 50 mM sodium phosphate buffer at pH 6.0, 2 mM SDS and variable amounts of enzyme (Table S7) at 25 °C. Molar extinction coefficients for the produced chromophores have already been reported^65^ and were used to calculate v_max_ and *k*_cat_ values by nonlinear regression, performed in the OriginPro 8 software. 7-8 substrate concentrations were chosen in regular intervals (Figs. S11-S16), limited by the solubility of the substrates and decreasing activity at high substrate concentrations. The parameters of the Michaelis-Menten equation (*K*_m_ and *k*_cat_) were fitted to the data measured during the kinetic assays. In the case of Phe260Gly and Leu244Arg the linear part of the Michaelis-Menten diagram of tyrosine was used to calculate the *K*_m_/*k*_cat_ ratio. Additionally, the acceptance of monophenolic (phenol, tyrosol, tyramine and *L*-tyrosine) and diphenolic substrates (catechol, 4-tert-butylcatechol, dopamine and *L*-DOPA) was tested by substrate scope assays. 1 mM of each substrate was mixed with 100 µg enzyme, 50 mM sodium phosphate buffer at pH 6.0 and 2 mM SDS in a final volume of 200 µl at 25 °C. The change of the color was detected visually, with a clearly visible change within 24 hours being construed as a positive outcome, and no change of color resulting in the substrate being evaluated as inactive. All assays were performed in triplicates. *jr*PPO1-wt was incubated for different periods of time to check if there is a time dependent inactivation due to the presence of SDS (Table S8). An analysis of variance with a post-hoc test (Tukey) was employed to analyse statistical significance of the kinetic parameters (Table S9).

### Molecular docking with *jr*PPO1 and the five mutants

Molecular docking was performed using AutoDock Vina^66^ to identify binding poses of monophenolic (tyramine and *L*-tyrosine) and diphenolic substrates (dopamine and *L*-DOPA) within the active site of *jr*PPO1-wt and the five mutants in order to structurally analyse substrate binding and to identify differences between the wildtype and the mutants. The crystal structure of *jr*PPO1 (wildtype, PDB entry 5CE9) was prepared for molecular docking by adding missing side chains using COOT^67^. The mutants of *jr*PPO1 were prepared by mutating the respective amino acids, which was also done with COOT. The gatekeeper (Phe260) and activity controller residues (Asn240 and Leu244) were defined as flexible residues and the exhaustiveness was set to 100. Structures of the substrates were obtained from the PDB and formatted into pdbqt files using AutoDockTools (ADT, v. 1.5.6)^66^, which specifies and samples all rotatable bonds and computes partial charges for the substrate structures. Binding poses were searched in a grid box of 12 × 12 × 12 Å^3^ (spacing = 1.0 Å) centered in-between the two copper ions of the active site. The docking settings (i.e. the grid box) were tested with the structure of TYR from *Bacillus megaterium* (*Bm*TYR) using *L*-tyrosine as a substrate. The resulting docking poses obtained from Autodock Vina applying our settings resembled almost perfectly the tyrosine pose found in the crystal structure of the *Bm*TYR-tyrosine complex (PDB entry 4P6R)^68^ indicating that the defined settings were suitable. Docking was performed with all protonation states of each substrate, i.e. protonated, semi-protonated and deprotonated (hydroxyl)phenyl group. For each target and substrate 20 poses were calculated. Upon docking the binding poses were evaluated by superimposing the docked substrate position with that of *L*-tyrosine from the *Bm*TYR-tyrosine structure. Poses that significantly deviated from the binding pose of *L*-tyrosine were flagged as’unreasonable’ poses.

## Supplementary information

Supplementary Information.
