# Supplementary material for: Conversion of walnut tyrosinase into a catechol oxidase by site directed mutagenesis
Source: Sci Rep. 2020 Feb 3;10:1659. doi: 10.1038/s41598-020-57671-x (PMC6997208; doi:10.1038/s41598-020-57671-x)
Supplement: Supplementary file 1 — Supplementary Information. [file 41598_2020_57671_MOESM1_ESM.docx]

**Supplementary Information**

**Conversion of walnut tyrosinase into a catechol oxidase by site directed mutagenesis**

Felix Panis,^[1]^ Ioannis Kampatsikas^[1]^, Aleksandar Bijelic^[1]^ & Annette Rompel*^[1]^

^1^ Universität Wien, Fakultät für Chemie, Institut für Biophysikalische Chemie, Althanstraße 14, 1090 Wien, Austria; https://www.bpc.univie.ac.at

*Correspondence to: [annette.rompel@univie.ac.at](mailto:annette.rompel@univie.ac.at)

**Table of content**

1. Supplementary Materials and Methods 2
2. Supplementary Tables 4
3. Supplementary Figures 10
4. References 35

**1. Supplementary Materials and Methods**

**Characterization of recombinantly expressed *jr*PPO1-wt.** For the characterization of recombinantly expressed, latent *jr*PPO1-wt pH and SDS optima were determined. To overcome the latency of *jr*PPO1-wt, SDS was used as an activator. SDS has extensively been used before and is widely accepted since it decreases the latency of PPOs without affecting the substrate preference ^1^. Contrary, the activation by acidic pH was reported to result in significantly weaker activation, making SDS the activation method of choice^2^. Complete activation of latent PPO is dependent on the SDS-concentration and the pH of the solution (1 mM substrate in 50 mM phosphate buffer). Different molarities of SDS have been reported for maximum activation of plant PPOs ranging from 0.35 mM^3^ to 4 mM^2^, while pH optima fluctuated from pH 4.5^4^ to pH 8.0^5^. Herein, the effects of increasing SDS concentrations and pH values were evaluated with the substrates tyramine and dopamine in order to determine the conditions that result in the highest diphenolase activity of *jr*PPO1-wt. For both substrates, the highest activity for *jr*PPO1-wt was observed at pH 6.0 at a final concentration of 2 mM SDS (Figure S17 and S18). These parameters were then applied to the following kinetic measurements for the *jr*PPO1-wt and the investigated mutants.

**Heterologous expression of *jr*PPO1-wt *via* auto-induction.** Auto-induction-medium (1.6 % tryptone, 1 % yeast extract, 0.5 % NaCl, 2 % lactose, 0.05 % glucose) was inoculated with an overnight culture grown in LB medium (10 g/l tryptone, 10 g/l NaCl, 5 g/l yeast extract), supplemented with 0.05 % glucose 30 minutes before inoculation. The expression batch was incubated at 37 °C and 240 rpm until the OD_600_ reached a value of 0.5. Subsequently, the samples were cooled to 20 °C, 0.5 mM CuSO_4_ were added and the culture was incubated at 20 °C and 240 rpm for 65 hours.

**Expression and characterization of the *jr*PPO1-wt variant Ile107Val.**

*jr*PPO1, as first described by Escobar et al. features Val in position 107^6^. In contrast, the crystal structure of *jr*PPO1 reported by Bijelic et al. exhibits an Ile in Position 107^7^. The sequence of *jr*PPO1 purified and characterised by Zekiri et al. was confirmed by nanoUHPLC–ESI-MS/MS of tryptic peptides, however, no peptide containing the amino acid in position 107 was found^8^. Therefore, it can only be speculated whether Ile or Val was present. In this study sequencing of the gene coding for *jr*PPO1-wt revealed Ile in position 107. To check if this exchange does influence the kinetic behavior of *jr*PPO1 we introduced Val in position 107 by site directed mutagenesis, yielding the mutant Ile107Val. Ile107Val was expressed as described for *jr*PPO1-wt and yielded simal amounts of active protein (Ile107Val: 40 mg/l, *jr*PPO1-wt: 38 mg/l).

pH and SDS optima were determined as described for *jr*PPO1-wt and Ile107Val showed a SDS- and pH profile similar to *jr*PPO1-wt and exhibits optima identical to *jr*PPO1-wt (pH = 6.0; SDS = 2 mM). Furthermore, Ile107Val was assessed kinetically. *K*_cat_ and *K*_m_ values were determined for dopamine leading to similar values in comparison to *jr*PPO1-wt (Table S10). Furthermore, CD-spectra of *jr*PPO1-wt, the mutant Ile107Val and the five investigated mutants were recorded and showed a highly similar CD-profile for all enzymes (Figure S25). Thus, the mutation Ile107Val does not influence the correct folding of *jr*PPO1.

**2. Supplementary Tables**

**Table S1.** Enzymes, organisms and UniProt identifiers of plant PPOs listed in Figure S1.

| **Enzyme** | **Organism** | **UniProt identifier** |
| --- | --- | --- |
| ***jr*PPO1** | *Juglans regia* | C0LU17 |
| ***am*AS1** | *Antirrhinum majus* | [Q9FRX6](https://www.uniprot.org/uniprot/Q9FRX6) |
| ***cg*AUS1** | *Coreopsis grandiflora* | [A0A075DN54](https://www.uniprot.org/uniprot/A0A075DN54) |
| ***Ib*CO** | *Ipomoea batatas* | [Q5ENY2](https://www.uniprot.org/uniprot/Q5ENY2) |
| ***Md*PPO1** | *Malus domestica* (Golden Delicious) | [A0A238GSN3](https://www.uniprot.org/uniprot/A0A238GSN3) |
| ***Md*PPO2** | *Malus domestica* (Golden Delicious) | [A0A238GSS3](https://www.uniprot.org/uniprot/A0A238GSS3) |
| ***Md*PPO3** | *Malus domestica* (Golden Delicious) | [A0A238GT27](https://www.uniprot.org/uniprot/A0A238GT27) |
| ***Sl*PPO1** | *Solanum lycopersicum* | [A0A3P3ZL83](https://www.uniprot.org/uniprot/A0A3P3ZL83) |
| ***Sl*PPO2** | *Solanum lycopersicum* | [A0A3P3ZKL1](https://www.uniprot.org/uniprot/A0A3P3ZKL1) |
| ***To*PPO-1** | *Tarraxacum officinale* | [B2BX66](https://www.uniprot.org/uniprot/B2BX66) |
| ***To*PPO-2** | *Tarraxacum officinale* | [B3WFP2](https://www.uniprot.org/uniprot/B3WFP2) |
| ***To*PPO-3** | *Tarraxacum officinale* | [G4U402](https://www.uniprot.org/uniprot/G4U402) |
| ***To*PPO-4** | *Tarraxacum officinale* | [G4U403](https://www.uniprot.org/uniprot/G4U403) |
| ***To*PPO-5** | *Tarraxacum officinale* | [G4U404](https://www.uniprot.org/uniprot/G4U404) |
| ***To*PPO-6** | *Tarraxacum officinale* | [I7HUF2](https://www.uniprot.org/uniprot/I7HUF2) |
| ***To*PPO-7** | *Tarraxacum officinale* | [U3UB86](https://www.uniprot.org/uniprot/U3UB86) |
| ***To*PPO-8** | *Tarraxacum officinale* | [U3UB68](https://www.uniprot.org/uniprot/U3UB68) |
| ***To*PPO-9** | *Tarraxacum officinale* | [U3UB71](https://www.uniprot.org/uniprot/U3UB71) |
| ***To*PPO-10** | *Tarraxacum officinale* | [U6C8D7](https://www.uniprot.org/uniprot/U6C8D7) |
| ***To*PPO-11** | *Tarraxacum officinale* | [U3UB75](https://www.uniprot.org/uniprot/U3UB75) |
| ***Vv*PPOcs-3** | *Vitis vinifera* (Cabernet Sauvignon) | [A0A0S2SVY3](https://www.uniprot.org/uniprot/A0A0S2SVY3) |
| ***Vv*PPOg** | *Vitis vinifera* (Grenache) | [P93622](https://www.uniprot.org/uniprot/P93622) |

**Table S2.** Heterologous expression of plant PPOs.

| **Enzyme** | **Affinity tag** | **Expression strain** | **T (°C)** | **Yield (mg)** | **Reference** |
| --- | --- | --- | --- | --- | --- |
| PPO  (*Camellia sinensis*) | 6xHis | BL21 (DE3) | 37 | insoluble | ^9^ |
| PPOs  (*Trifolium pratense*) | 6xHis | BL21 (DE3) RIL | 37 | insoluble | ^10^ |
| PPO  (*Malus domestica*) | GST | XL-1 blue | 37 | insoluble | ^11^ |
| *Cs*PPO-1 and 2  (*Camellia sinensis*) | GST | BL21 (DE3) | 37 | insoluble | ^12^ |
| PPO-6  (*Taraxacum officinale*) | Strep-tag II | Rosetta™ 2(DE3) /pLysSRARE2 | 26 | 0.5-2 | ^13^ |
| *To*PPO-2/6  (*Taraxacum officinale*) | Strep-tag II | Rosetta™ 2(DE3) /pLysSRARE2 | 26 | 0.7-3.9 | ^14^ |
| *cg*AUS1  (*Coreopsis grandiflora*) | 6xHis | Top 10 | 37 | 5-6 | ^15^ |
| *To*PPO-2  (*Taraxacum officinale*) | Strep-tag II | Rosetta™ 2(DE3) /pLysSRARE2 | 26 | 8.7±2.9 | ^16^ |
| *Md*PPO1-3  (*Malus domestica*) | GST | BL21 (DE3) | 20 | 26.5-225 | ^2^ |
| *jr*PPO1-wt  *(Juglans regia)* | GST | BL21 (DE3) | 20 | 38 | this work |

Heterologous expression of plant PPOs showing the variety of affinity tags, expression hosts and expression temperatures with the corresponding yields of soluble protein.

**Table S3.** Calculated and measured masses of *jr*PPO1-wt fragments after trypsin cleavage.

| ***jr*PPO1-wt fragment** | **Molecular mass (calculated)** | **Molecular mass (measured)** | **Δ/Da** |
| --- | --- | --- | --- |
| **Gly-3–Lys350** | 39932.63 | 39932.80 | +0.17 |
| **Gly-3–Arg348** | 39705.33 | 39705.00 | -0.33 |
| **Ala355–Gly503** | 16018.15 | 16018.14 | -0.01 |

**Table S4.** Primers used for the amplification of the wild-type *jr*PPO1 gene and the generation of the investigated mutants.

| **Primer** | **Sequence** |
| --- | --- |
| ***jr*PPO1-wt ORF*** | fwd: 5´*agcggctcttcaatg*GATCCGGTATCCGCGCCG 3´ |
|  | rev: 5´*agcggctcttctccc*TCAACCGATAAGCACAATCTTGATCCC 3´ |
| **Phe260Gly** | fwd: 5´GAGAACATGGGGAACggCTACTCGGCCGGTAGAGATCCA 3´ |
|  | rev: 5´GATATTAGGTTGGGTGTCGTCACCGGTCC 3´ |
| **Asn240Lys** | fwd: 5´CACTCCCCACAAgAATATCCACCTATGGACCGG 3´ |
|  | rev: 5´CTCTCGATTGTGCCAGCACCTGGG 3´ |
| **Leu244Arg** | fwd: 5´CCCACAATAATATCCACcgATGGACCGGTGACGAC 3´ |
|  | rev: 5´GAGTGCTCTCGATTGTGCCAGCACC 3´ |
| **Asn240Lys/**  **Leu244Arg** | fwd: 5´CACTCCCCACAAgAATATCCACCgATGGACCGGTGAC 3´ |
|  | rev: 5´CTCTCGATTGTGCCAGCACCTGGG 3´ |
| **Asn240Lys/**  **Leu244Thr** | fwd: 5´CACTCCCCACAcTAATATCCACCgcTGGACCGGTGACGACACC 3´ |
|  | rev: 5´CTCTCGATTGTGCCAGCACCTGGG 3´ |

Primers targeting the gene coding the latent form of *jr*PPO1-wt (*). The nucleotides in italics were used for cloning the amplicon. In the primers used for the generation of the mutants the substituted nucleotides are depicted as small letters.

**Table S5.** Comparison of the binding affinity calculated by AutoDock Vina and the experimentally determined *K*_m_ values.

| **Target** | **Substrate** | ***K*_m_ (mM)** | **Affinity (kcal/mol)^[a]^** | **No. of ‘reasonable’ poses^[b]^** |
| --- | --- | --- | --- | --- |
| **Wild type** | tyramine  *L*-tyrosine  dopamine  *L*-DOPA | 0.5  1.4  0.8  18.1 | -6.4  -6.4  -6.4  -6.7 | 3/20  3/20  2/20  2/20 |
| **Phe260Gly** | tyramine  *L*-tyrosine  dopamine  *L*-DOPA | 3.1  n.a.*  5.9  10.5 | -4.9  -5.0  -5.4  -5.9 | 3/20  2/20  3/20  2/20 |
| **Asn240Lys** | tyramine  *L*-tyrosine  dopamine  *L*-DOPA | 8.2  n.a.*  19.8  16.7 | -6.6  -7.2  -6.9  -7.1 | 3/20  4/20  2/20  4/20 |
| **Leu244Arg** | tyramine  *L*-tyrosine  dopamine  *L*-DOPA | 2.7  n.a.*  2.4  4.2 | -6.4  -7.5  -6.5  -7.1 | 4/20  4/20  3/20  2/20 |
| **Asn240Lys/Leu244Arg** | tyramine  *L*-tyrosine  dopamine  *L*-DOPA | n.a.*  n.a.*  6.8  16.5 | -7.3  -7.3  -7.4  -7.3 | 4/20  4/20  3/20  3/20 |
| **Asn240Thr/Leu244Arg** | tyramine  *L*-tyrosine  dopamine  *L*-DOPA | n.a.*  n.a.*  6.6  13.1 | -6.6  -7.0  -6.9  -6.7 | 3/20  3/20  3/20  2/20 |

^[a]^ binding affinity calculated by AutoDock Vina for the most ‘reasonable’ binding pose.

^[b]^ This number indicates how many of the 20 generated poses were defined as ‘reasonable’.

* represents samples that were active but could not be measured due to extensively reduced reactivity and increased *K*_m_ values in combination with limited substrate solubility

**Table S6.** Ionic strength of buffers used for investigating the pH optimum of *jr*PPO1-wt.

| **Citrate buffer** | | **Phosphate buffer** | |
| --- | --- | --- | --- |
| **pH** | **Ionic strength (mM)** | **pH** | **Ionic strength (mM)** |
| **3.0** | 0.050 | **6.0** | 0.070 |
| **3.5** | 0.061 | **6.5** | 0.083 |
| **4.0** | 0.078 | **7.0** | 0.104 |
| **4.5** | 0.105 | **7.5** | 0.123 |
| **5.0** | 0.136 | **8.0** | 0.132 |
| **5.5** | 0.174 |  |  |

**Table S7.** Amount of enzyme (µg) used for determining kinetic parameters of *jr*PPO1-wt and the five mutants.

| **Enzyme** | **tyramine** | **tyrosine** | **dopamine** | ***L*-DOPA** |
| --- | --- | --- | --- | --- |
| ***jr*PPO1-wt** | 0.85 | 4.23 | 1.33 | 0.89 |
| **Phe260Gly** | 11.1 | 99.0 | 0.83 | 1.11 |
| **Asn240Lys** |  |  | 5.20 | 18.2 |
| **Leu244Arg** | 3.58 | 17.7 | 0.90 | 1.79 |
| **Asn240Lys/Leu244Arg** |  |  | 154 | 164 |
| **Asn240Thr/Leu244Arg** |  |  | 7.60 | 38.0 |

**Table S8.** Time dependent inactivation of *jr*PPO1-wt by SDS.

| **Incubation time (min)** | **Rel. activity (%)** |
| --- | --- |
| 0 | 100 ± 0.72 |
| 10 | 97 ± 0.60 |
| 30 | 92 ± 0.93 |
| 120 | 69 ± 1.1 |
| 300 | 58 ± 1.02 |

*jr*PPO1-wt was incubated with 2mM SDS for different periods of time in 50 mM sodium phosphate buffer at 25 °C. Activities were measured using 1mM tyramine as a substrate. Measurements were performed in triplicates.

**Table S9.** Statistical analysis (Tukey) of kinetic parameter.

| **Enzyme pairs** | **tyramine *k*_cat_** | **tyramine *K*_m_** | **dopamine *k*_cat_** | **dopamine *K*_m_** | ***L*-DOPA *k*_cat_** | ***L*-DOPA *K*_m_** |
| --- | --- | --- | --- | --- | --- | --- |
| **A vs B** | 0.001 | 0.001 | 0.001 | 0.679 | 0.001 | 0.020 |
| **A vs C** | 0.001 | 0.001 | 0.001 | 0.001 | 0.001 | 0.001 |
| **A vs D** | 0.001 | 0.001 | 0.001 | 0.005 | 0.001 | 0.522 |
| **A vs E** |  |  | 0.001 | 0.001 | 0.001 | 0.001 |
| **A vs F** |  |  | 0.001 | 0.001 | 0.001 | 0.001 |
| **B vs C** | 0.427 | 0.001 | 0.001 | 0.001 | 0.011 | 0.001 |
| **B vs D** | 0.001 | 0.321 | 0.001 | 0.052 | 0.837 | 0.001 |
| **B vs E** |  |  | 0.001 | 0.001 | 0.005 | 0.001 |
| **B vs F** |  |  | 0.001 | 0.001 | 0.005 | 0.246 |
| **C vs D** | 0.001 | 0.001 | 0.001 | 0.001 | 0.002 | 0.001 |
| **C vs E** |  |  | 0.045 | 0.001 | 0.899 | 0.851 |
| **C vs F** |  |  | 0.238 | 0.001 | 0.899 | 0.065 |
| **D vs E** |  |  | 0.001 | 0.001 | 0.001 | 0.001 |
| **D vs F** |  |  | 0.001 | 0.001 | 0.001 | 0.001 |
| **E vs F** |  |  | 0.889 | 0.899 | 0.899 | 0.010 |

p-values of Tukey test. Values < p = 0.05 represent statistical significance between two parameters. A = *jr*PPO1-wt, B = Phe260Gly, C = Asn240Lys, D = Leu244Arg, E = Asn240Lys/Leu244Arg and F = Asn240Thr/Leu244Arg.

**Table S10.** *k*_cat_ and *K*_m_ values of Ile107Val and *jr*PPO1-wt determined for dopamine.

| **Enzyme** | ***k*_cat_** | ***K*_m_** | ***k*_cat_/*K*_m_** |
| --- | --- | --- | --- |
| **Ile107Val** | 90.7 ± 3.9 | 0.742 ± 0.067 | 123 ± 12 |
| ***jr*PPO1-wt** | 92.5 ± 7.8 | 0.75 ± 0.13 | 123 ± 24 |

**3. Supplementary Figures**


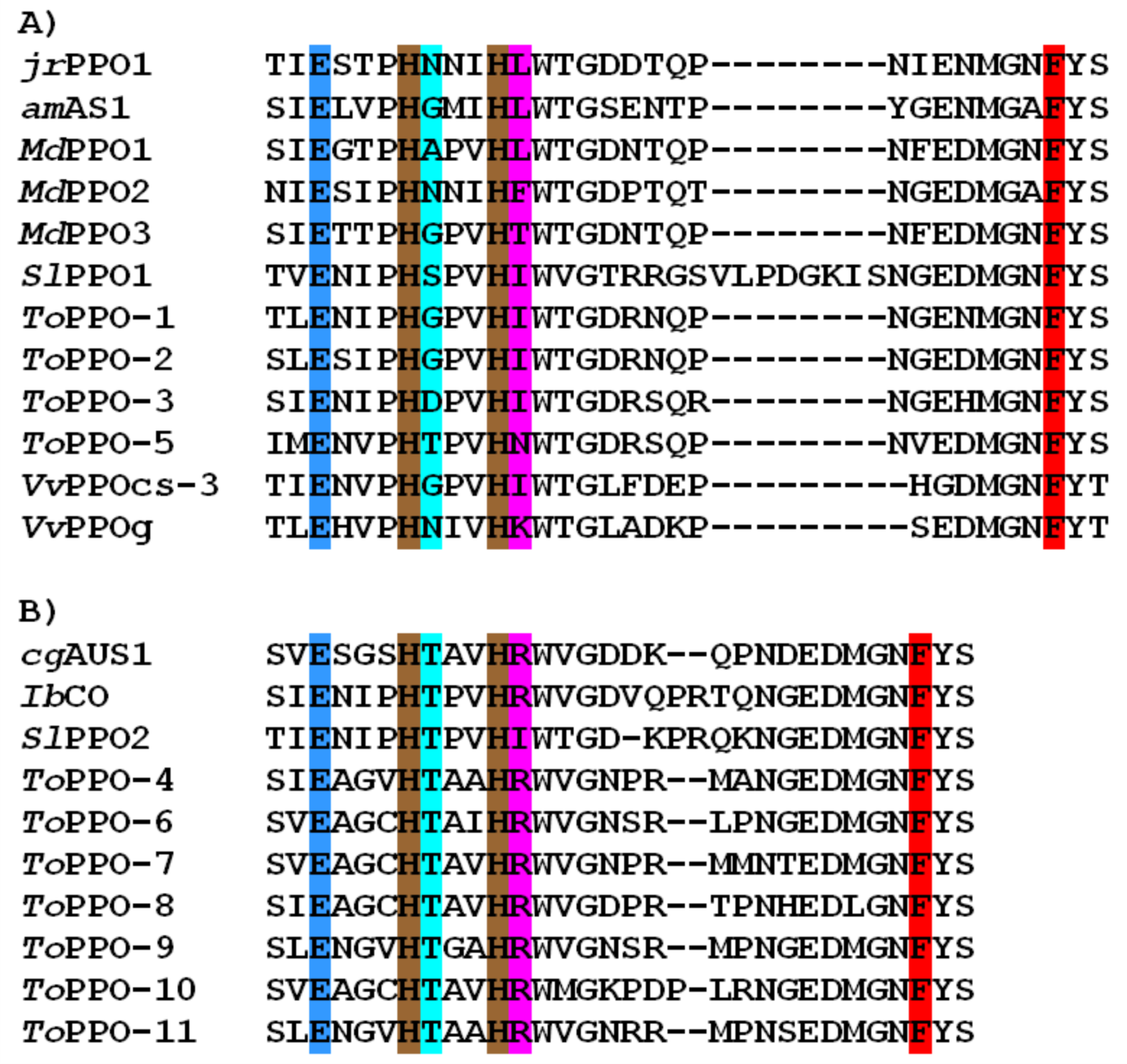


**Figure S1. Alignment of plant PPO sequences around the 1^st^ and 2^nd^ activity controller.** A) Tyrosinase sequences, B) Catechol oxidase sequences. Highlighted in blue: conserved glutamic acid, brown: His_B1_ (left) and His_B2_ (right), cyan: 1^st^ activity controller, magenta: 2^nd^ activity controller, red: gate keeper residue. UniProt identifiers of all enzymes are listed in Table S1.

**
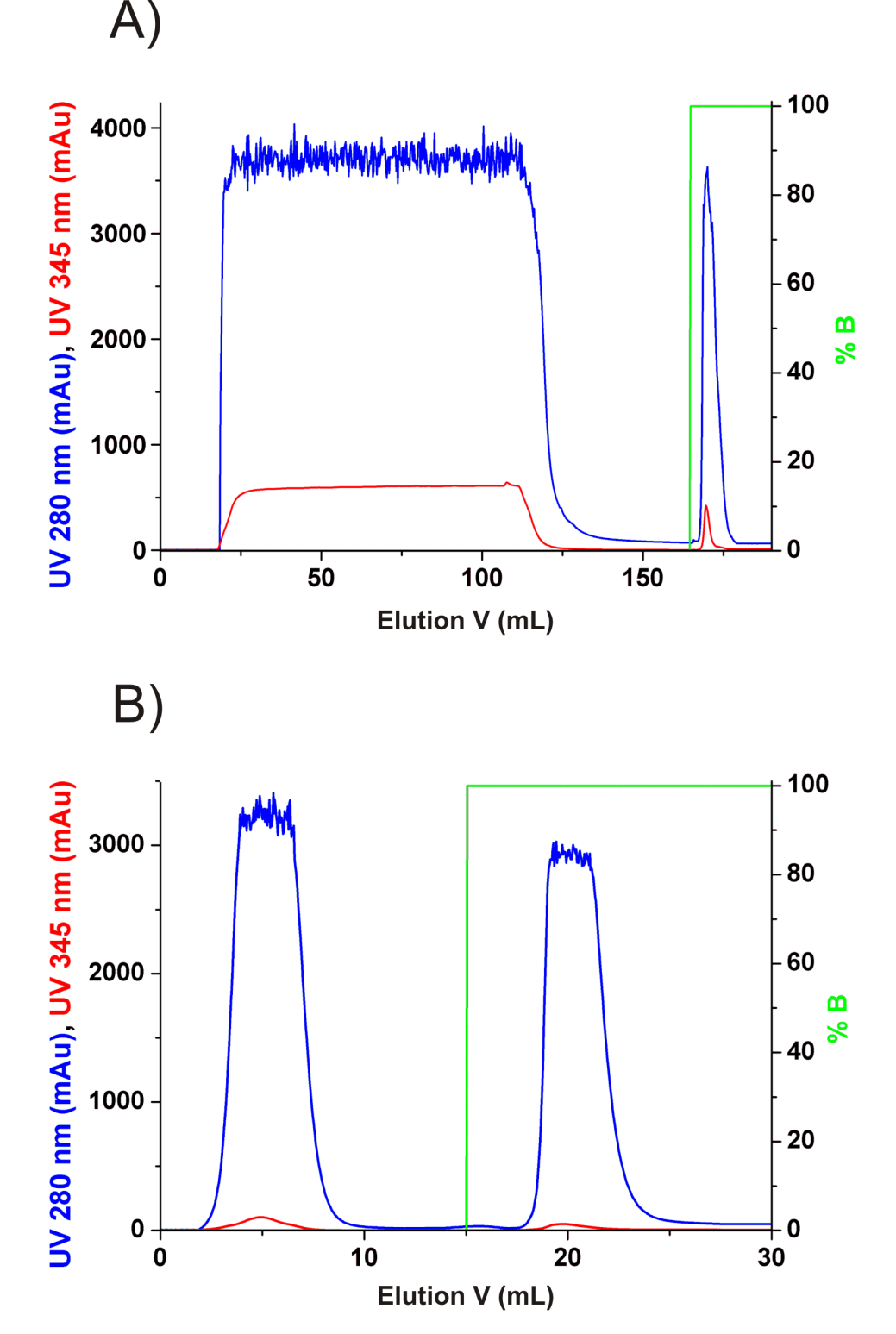
**

**Figure S2. Chromatogram of the 1^st^ and 2^nd^ GST-affinity purification of GST-*jr*PPO1-wt.** **A:** Lysate was applied to the column and the trapped protein was eluted with elution buffer (200 mM NaCl, 50 mM Tris-HCl, pH 7.8, 10 mM reduced glutathione). % B represents the concentration of the elution buffer. **B:** The absorption peak on the left side corresponds to the cleaved *jr*PPO1-wt, while the peak to the right corresponds to the GST-tagged protease and the GST-tag cleaved off the target PPO. % B represents the concentration of the elution buffer (200 mM NaCl, 50 mM Tris-HCl, 10 mM reduced glutathione, pH 7.8).

**

**

**Figure S3.** Positive mode ESI-LTQ-Orbitrap Velos of the latent, purified *jr*PPO1-wt after treatment with trypsin. Two different species of the catalytically active domain indicate two different cleavage sites. The first species corresponds to the fragment obtained upon cleavage between Lys350-Lys351, while the second species corresponds to cleavage between Arg348-Val349. The calculated and measured masses of the protein fragments are listed in the Table S4.

**
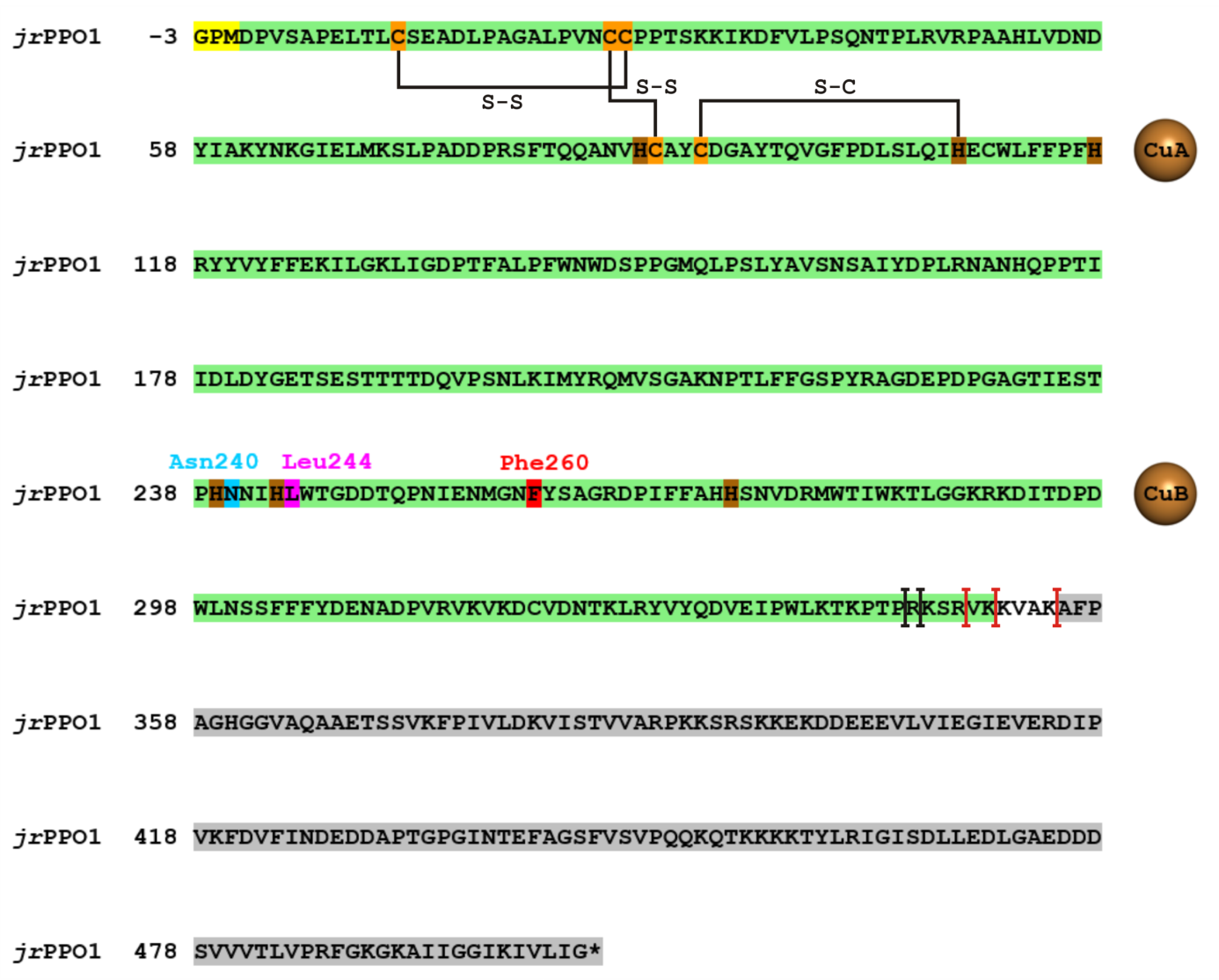
**

**Figure S4. Sequence of *jr*PPO1-wt.** The sequence of the recombinantly expressed *jr*PPO1-wt features three additional amino acids (Gly-Pro-Met) at the N-terminus (yellow). The three amino acids are part of the expression vector. The active domain is highlighted in green, the grey part marks the C-terminal domain. The unmarked region represents the proteolytically cleaved part between the two main domains. The 1^st^ activity controller residue is marked cyan, the 2^nd^ activity controller residue magenta and the blocker residue red. Copper coordinating histidines are highlighted in brown, the cysteins involved in the formation of disulfide bonds and the thioether bridge are highlighted in orange. Black lines (**
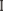
** ) indicate the main natural cutting sites^8^, red lines (**
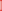
** ) mark the trypsin cutting sites as determined by ESI-MS in this study.

**
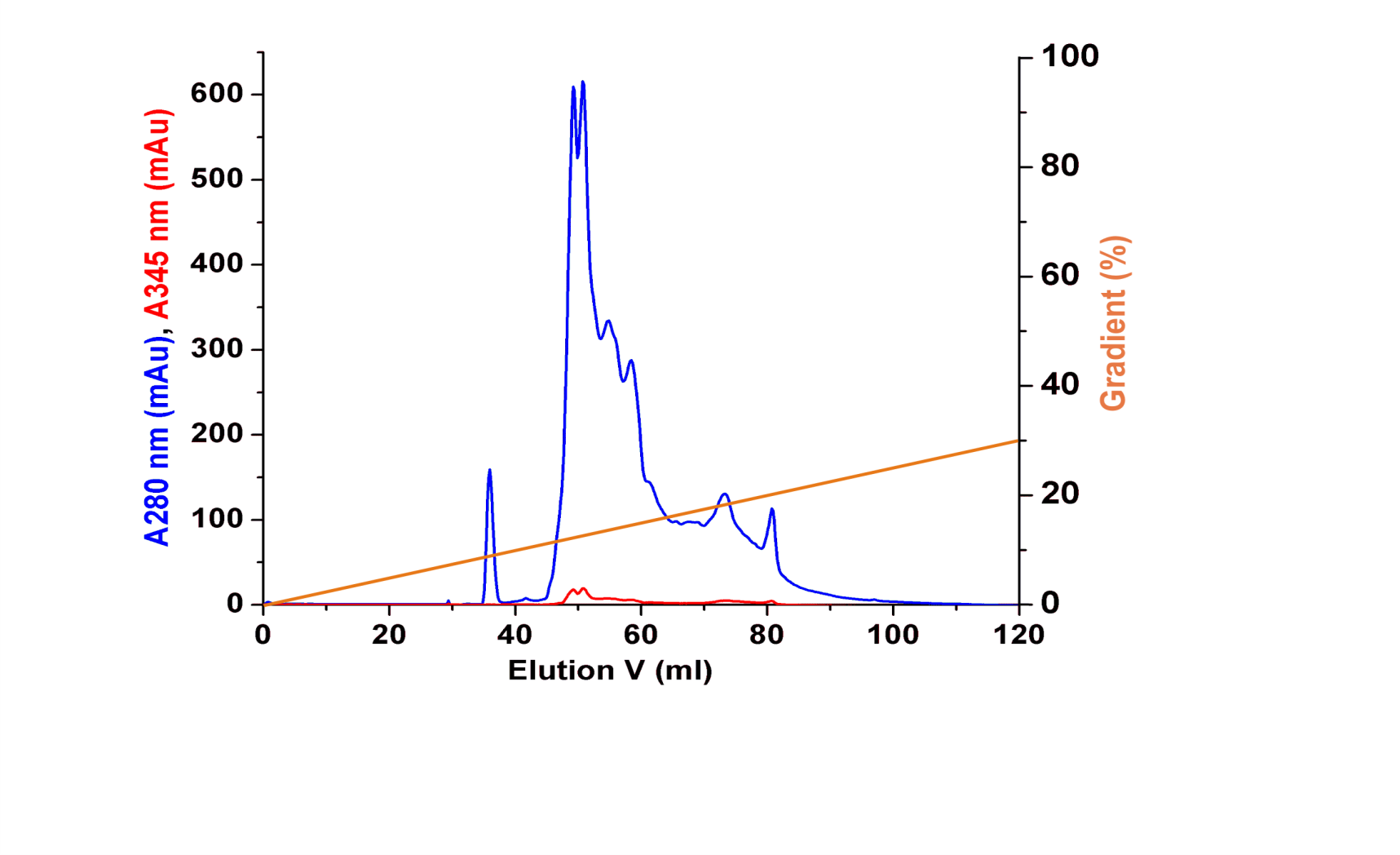
Figure S5. Chromatogram of the purification of the *jr*PPO1-wt after trypsin digestion *via* ion-exchange chromatography (Mono Q 5/50 GL).** The column was equilibrated using 10 mM Tris-HCl pH 7.8. 10 mg *jr*PPO1-wt (in 10 mM Tris-HCl pH 7.8) were applied and eluted using a linear gradient of 0 – 250 mM NaCl in 10 mM Tris-HCl, pH 7.8.





**Figure S6. Chromatogram of *jr*PPO1-wt purification after trypsin digestion *via* size-exclusion chromatography (Superdex 200 increase 10/300 GL).** 10 mg protein (in 150 µl 200 mM NaCl, 50 mM Tris-HCl, pH 7.8) were separated using a 200 mM NaCl, 50 mM Tris-HCl, pH 7.8 running buffer.


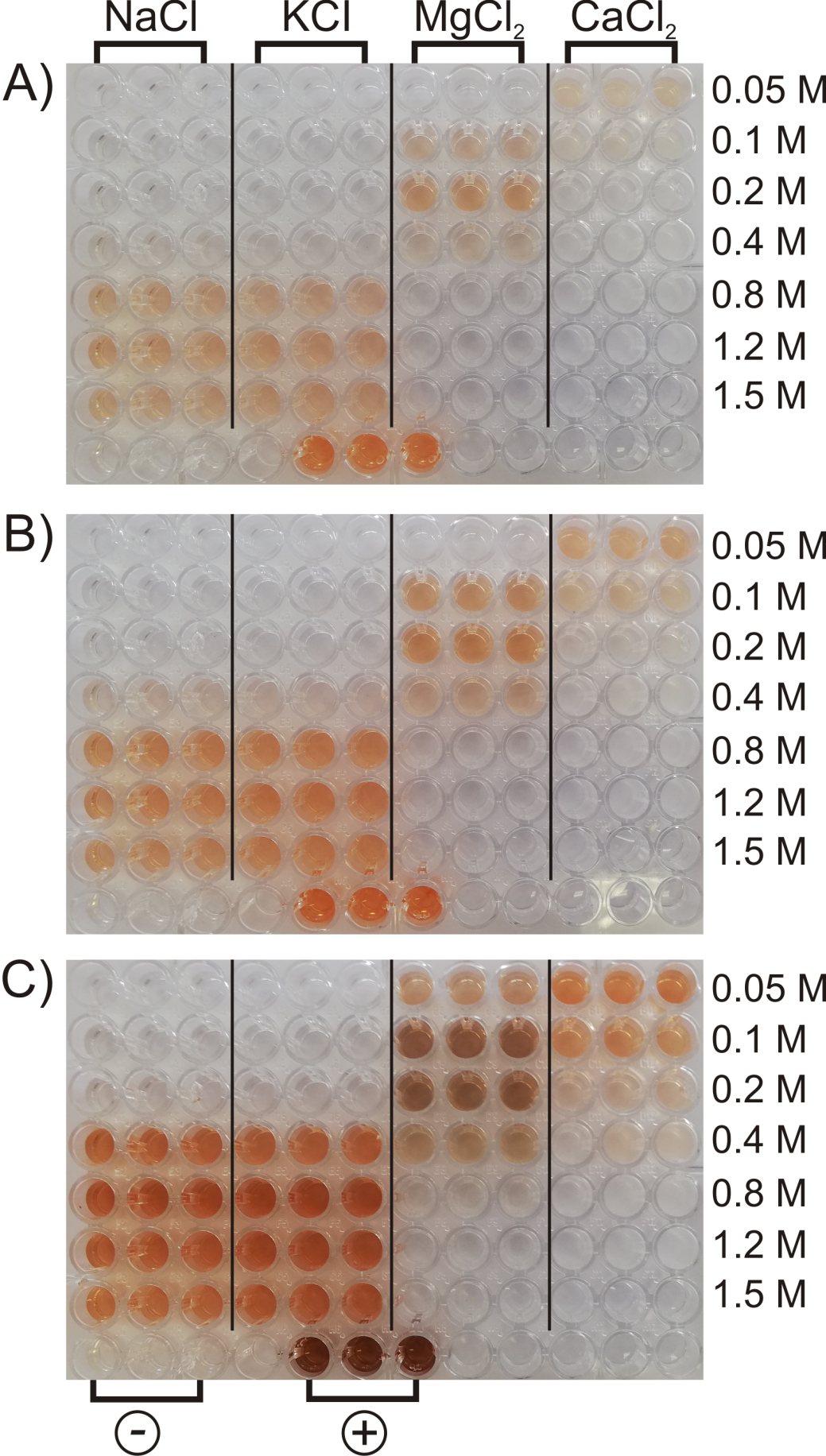


**Figure S7**. Activation of *jr*PPO1-wt with different molarities of salts after different periods of time: A = 2 minutes, B = 5 minutes, C = 10 minutes. 1 mM dopamine was mixed with 1 µg enzyme in 200 µl 50 mM phosphate buffer pH = 6.0. All assays were performed in triplicates. Wells 1-3 in the bottom row represent the negative control (no salt or SDS added as activator), wells 5-7 in the bottom row represent the positive control (2 mM SDS added as activator).

**
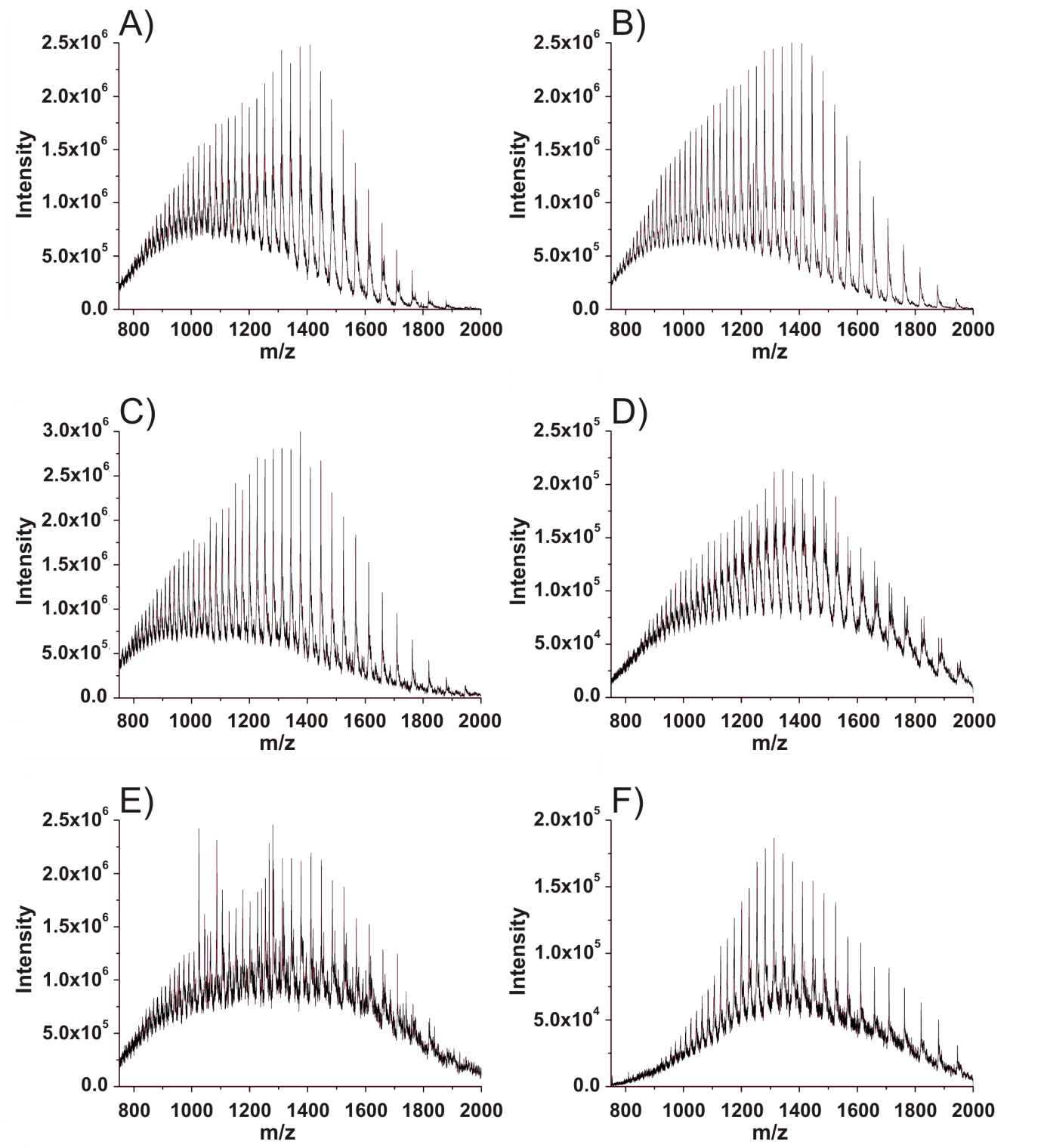
Figure S8. Mass spectra of *jr*PPO1-wt and the five mutants.** A = *jr*PPO1-wt, B = Phe260Gly, C = Asn240Lys, D = Leu244Arg, E = Asn240Lys/Leu244Arg, F = Asn240Thr/Leu244Arg. Entire mass spectrum of acidified recombinant latent enzymes. The calculated and measured masses of the proteins are listed in Table 2 of the main manuscript.

**
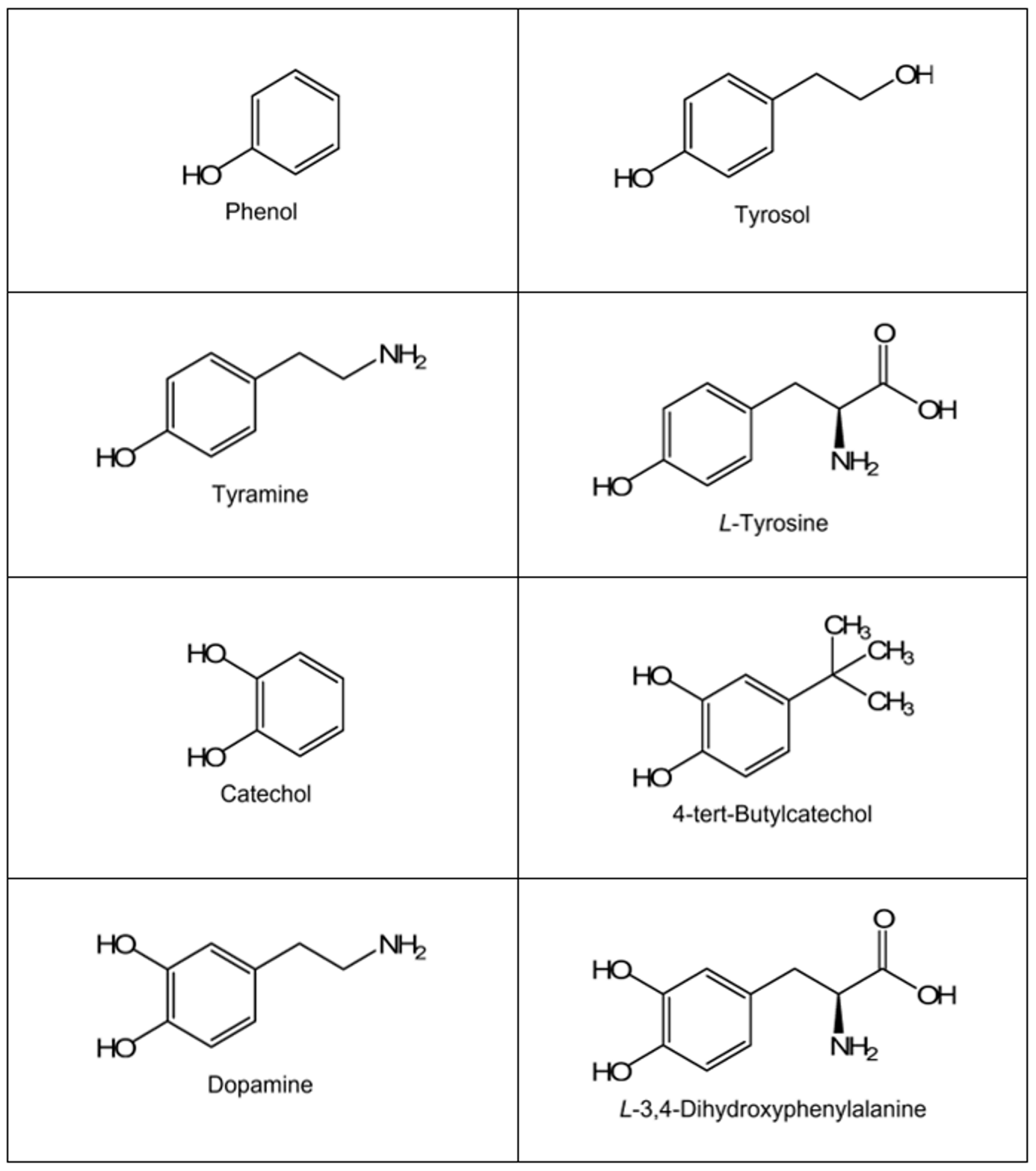
Figure S9. Structures of monophenolic and diphenolic substrates.**


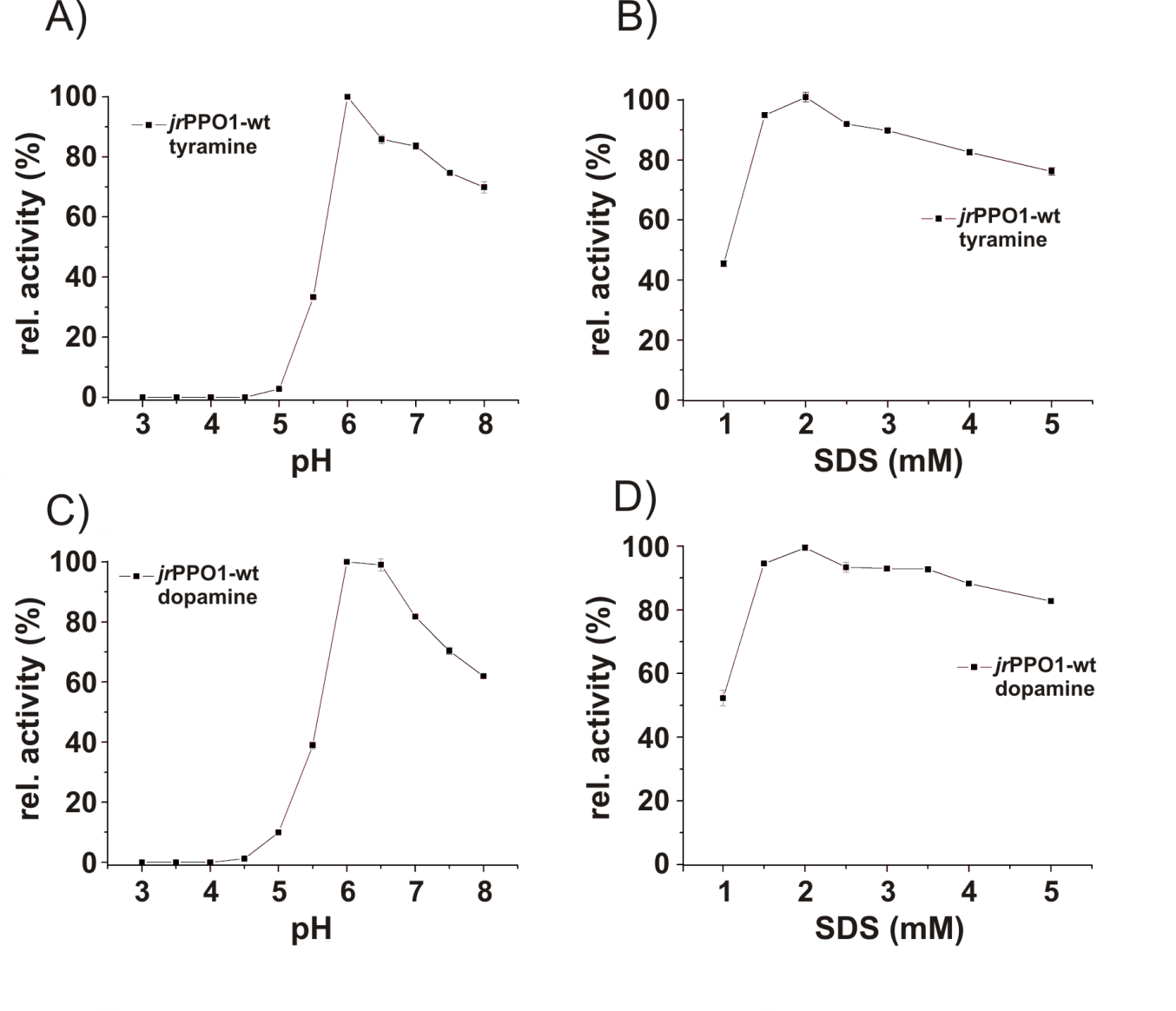


**Figure S10. pH and SDS optima for activity induction of *jr*PPO1-wt with 1 mM tyramine (A&B) or dopamine (C&D).** Activities are plotted in relation to the maximum activity. The error bars indicate ± one standard deviation. Detailed information about the experimental setup is provided in the materials and methods section.
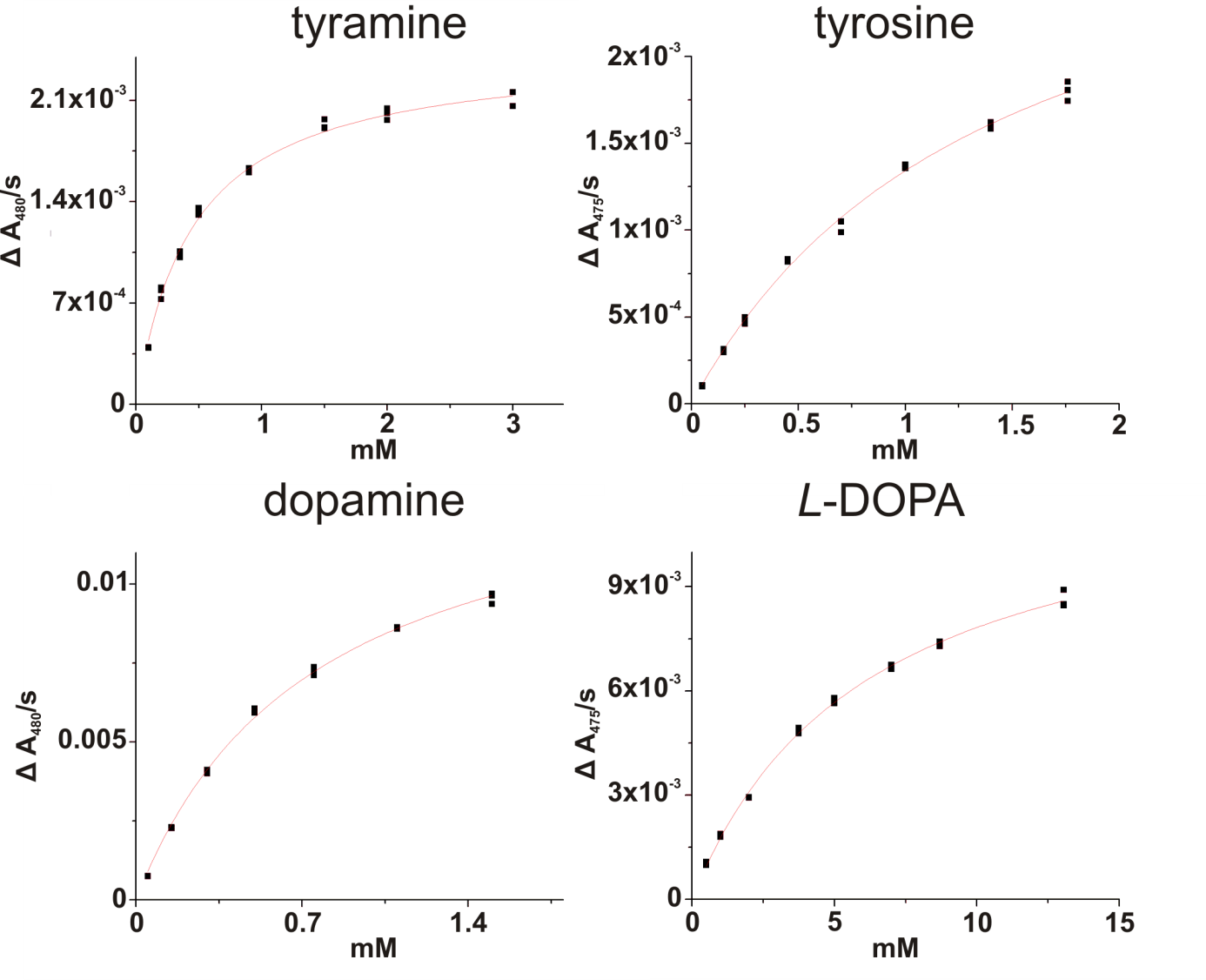
**Figure S11. Non-liner curve fitting of data points measured for *jr*PPO1-wt during kinetic assays.** Triplets were fitted using the Hill-equation and the least squares method build in the OriginPro 8 software.


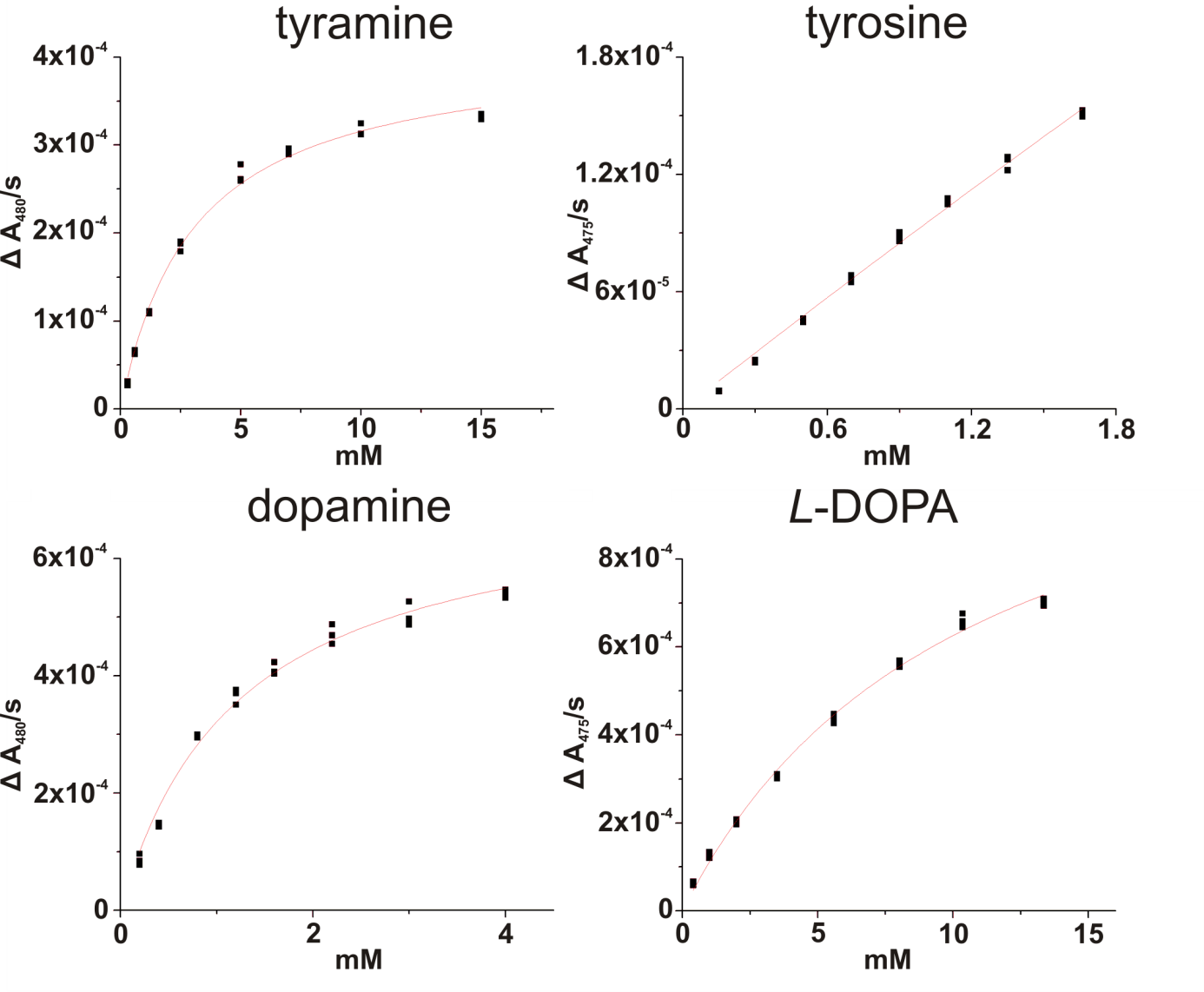


**Figure S12. Non-liner curve fitting of data points measured for Phe260Gly during kinetic assays.** Triplets were fitted using the Hill-equation and the least squares method build in the OriginPro 8 software.


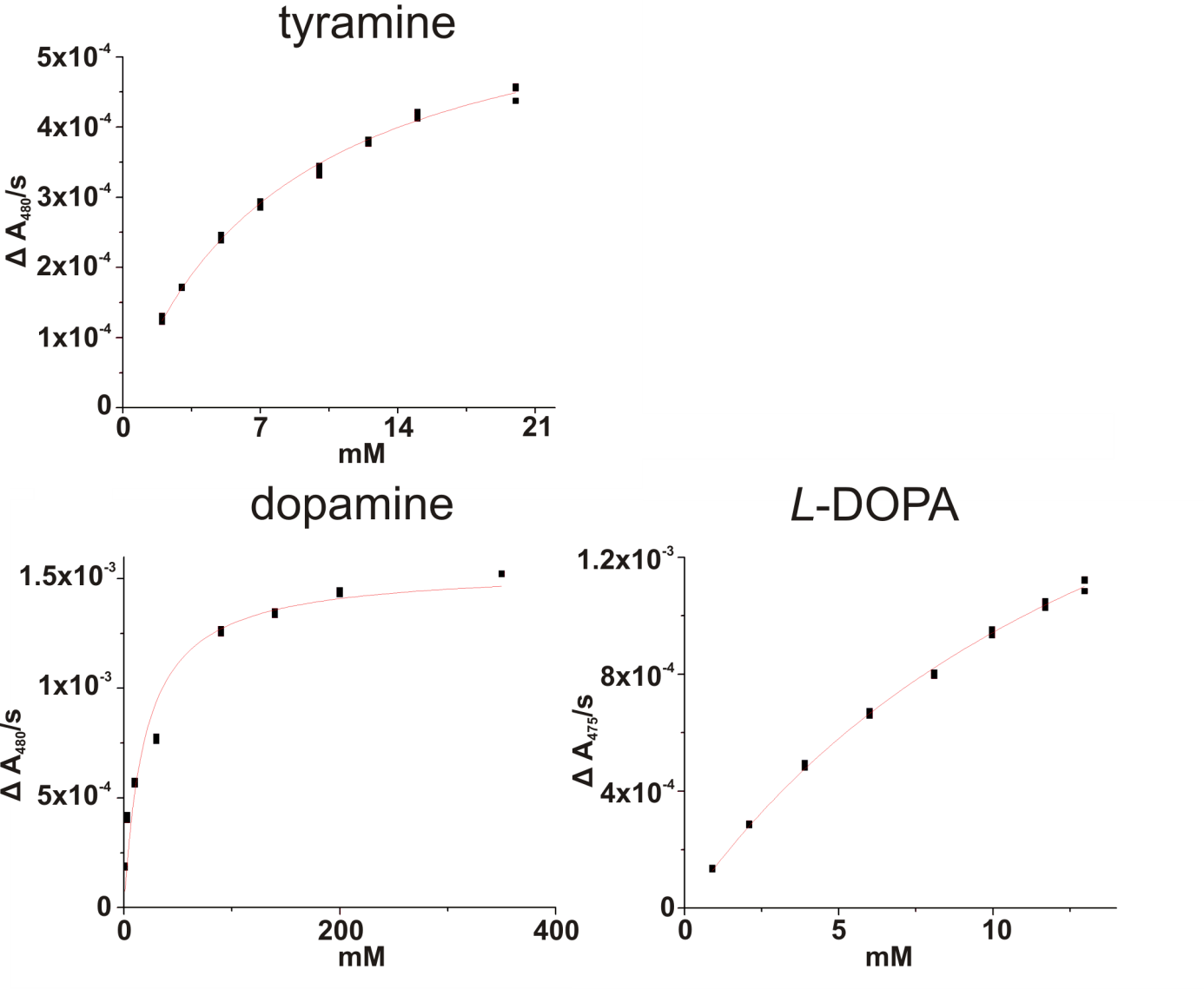


**Figure S13. Non-liner curve fitting of data points measured for Asn240Lys during kinetic assays.** Triplets were fitted using the Hill-equation and the least squares method build in the OriginPro 8 software.


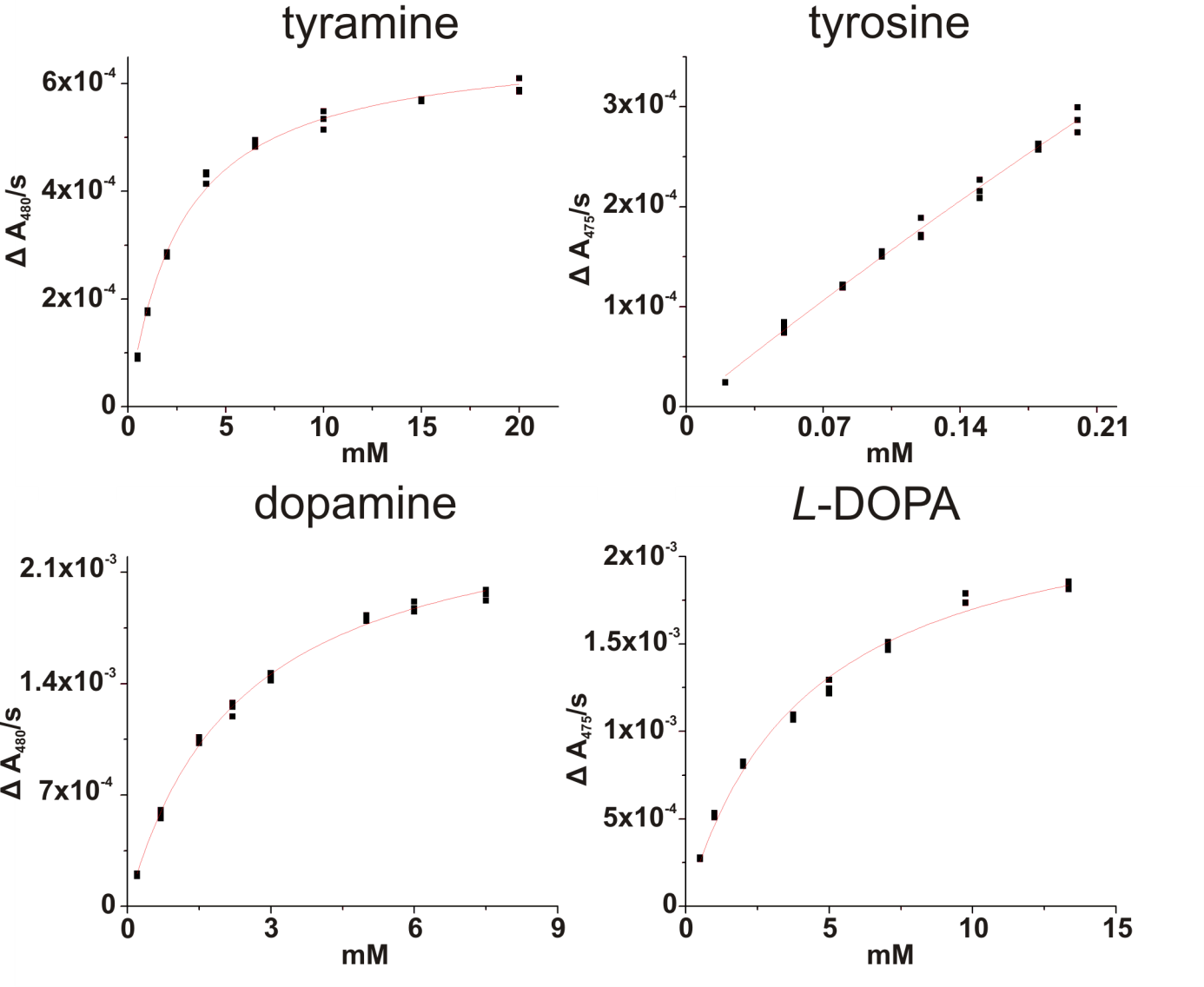


**Figure S14. Non-liner curve fitting of data points measured for Leu244Arg during kinetic assays.** Triplets were fitted using the Hill-equation and the least squares method build in the OriginPro 8 software.


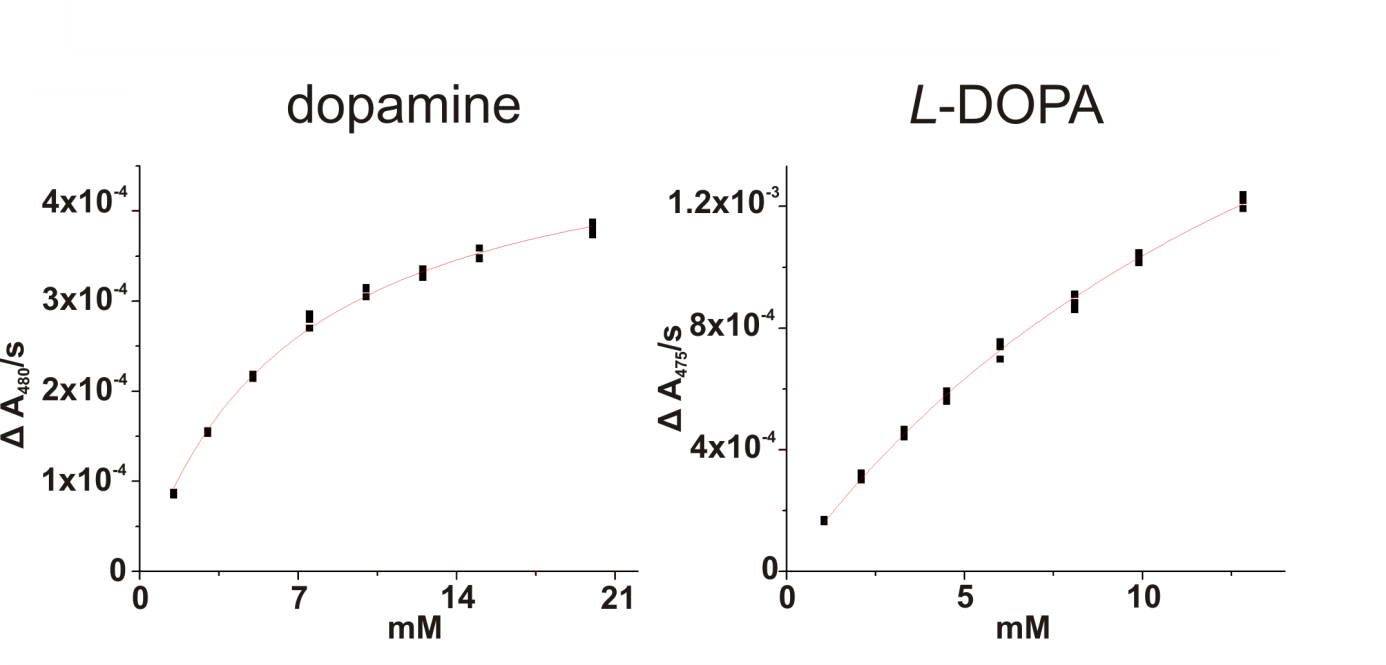


**Figure S15. Non-liner curve fitting of data points measured for Asn240Lys/Leu244Arg during kinetic assays.** Triplets were fitted using the Hill-equation and the least squares method build in the OriginPro 8 software.

**
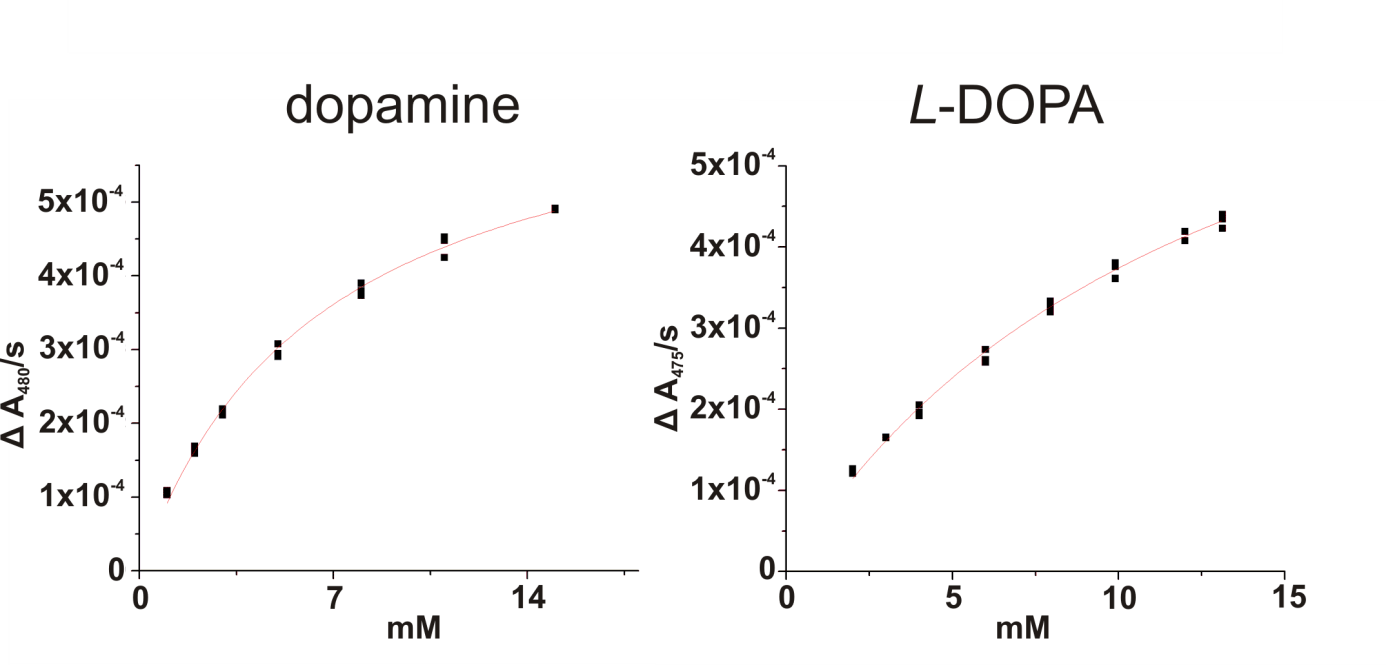
Figure S16. Non-liner curve fitting of data points measured for Asn240Thr/Leu244Arg during kinetic assays.** Triplets were fitted using the Hill-equation and the least squares method build in the OriginPro 8 software.


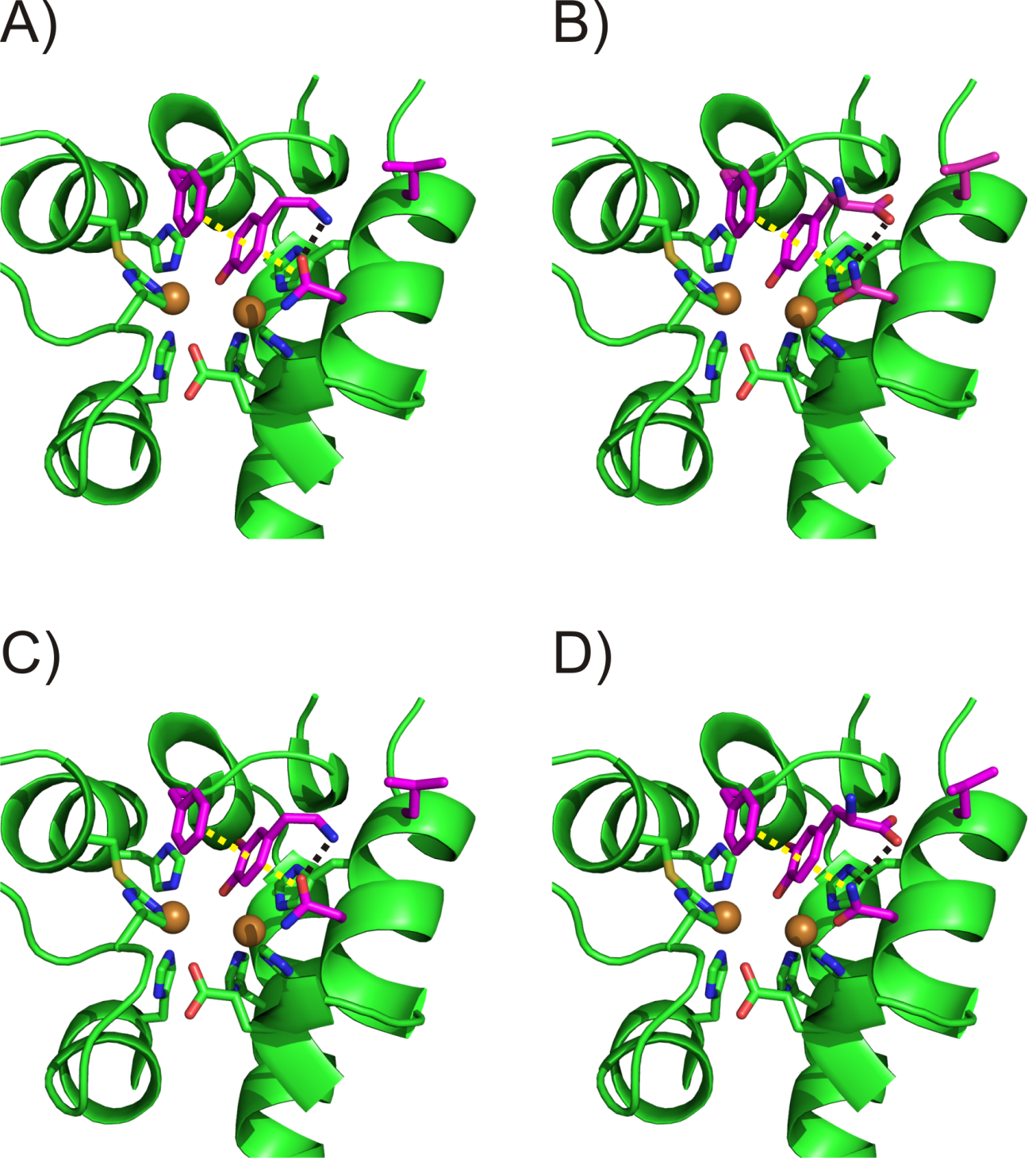


**Figure S17. Substrate binding to the wildtype of *jr*PPO1 according to docking studies**. Binding of A) tyramine, B) *L*-tyrosine, C) dopamine and D) *L*-DOPA. Each substrate is mainly stabilized by the Phe260-substrate-His243 π-stacking system (dashed yellow line). In addition, the 1^st^ activity controller Asn240 interacts with the tail of each substrate *via* hydrogen bonding (dashed black lines). The residues colored in magenta represent those that were defined as flexible residues during the docking process.


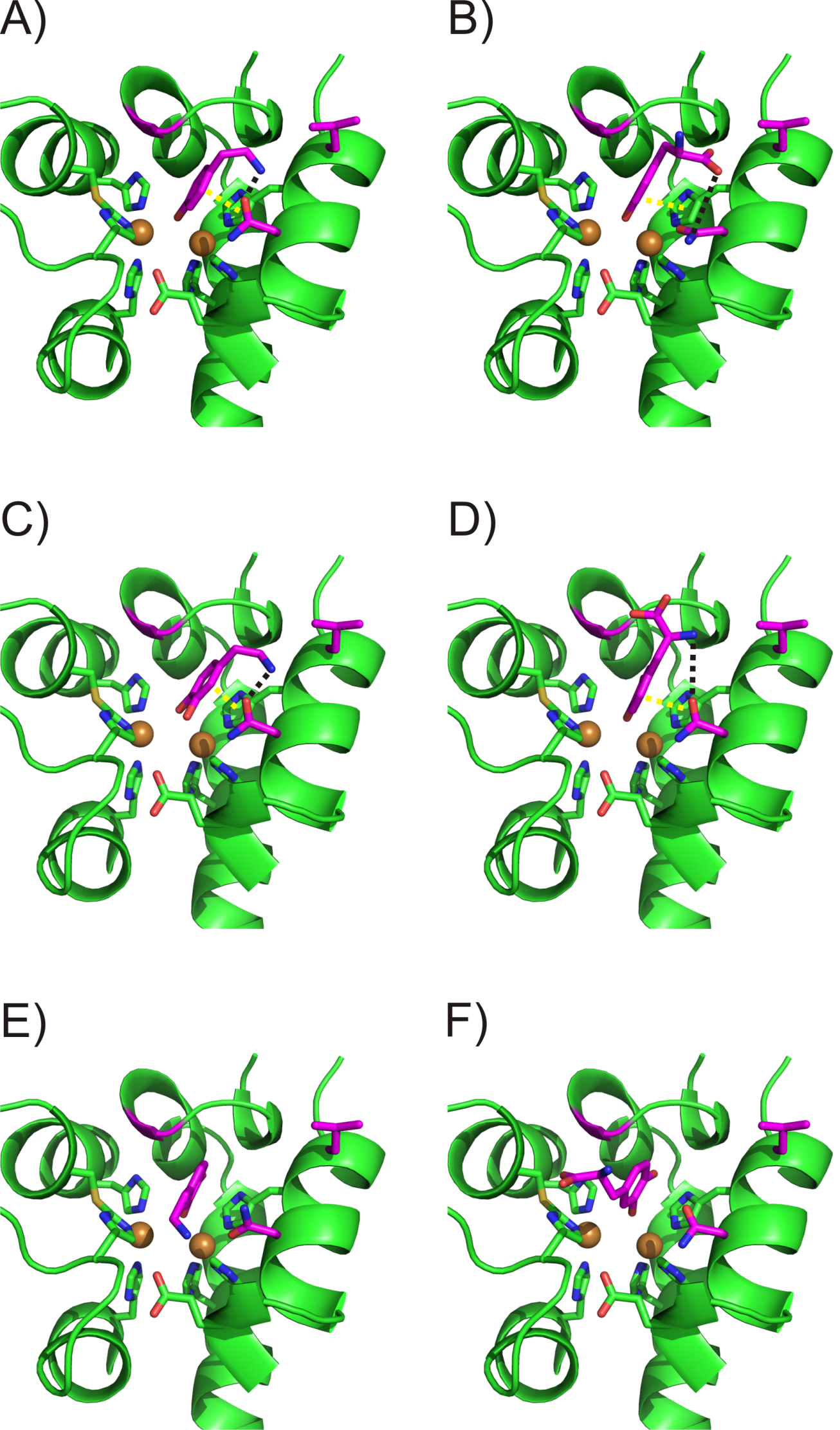


**Figure S18. Substrate binding to Phe260Gly according to docking**. Binding of A) tyramine, B) *L*-tyrosine, C) dopamine and D) *L*-DOPA in the most ‘reasonable’ manner. By comparing these poses with those of the *jr*PPO1-wt (Figure S17), it can be seen that the substrates even in their most ‘reasonable’ poses within Phe260Gly are still shifted and not well orientated within the active site. The best binding poses of E) dopamine and F) *L*-DOPA according to AutoDock show that the substrates are incorrectly positioned within the active site. Dopamine interacts with its amine group weakly with Asn240 but points with its dihydroxyphenyl group towards CuB. Similarly, *L*-DOPA is orientated towards CuB with its tail pointing into the direction of the bulk solvent. The residues colored in magenta represent those that were defined as flexible residues during the docking process.


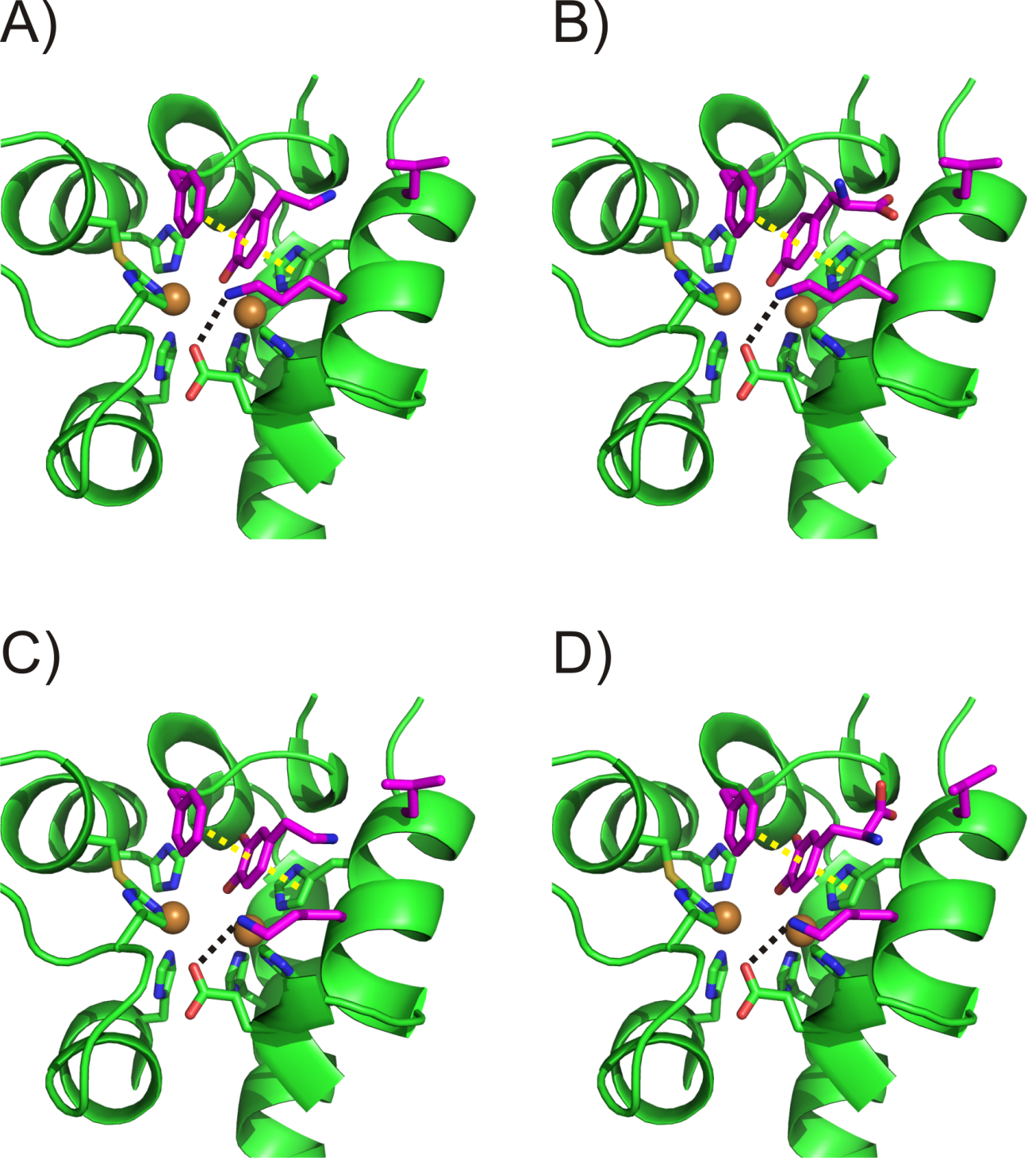


**Figure S19. Substrate binding to Asn240Lys according to docking**. Binding of A) tyramine, B) *L*-tyrosine, C) dopamine and D) *L*-DOPA. Each substrate is mainly stabilized by the Phe260-substrate-His243 π-stacking system (dashed yellow line). Lys240 is in each case in close proximity to Glu235 indicating the possibility of a strong ionic interaction between these residues (dashed black lines). The residues colored in magenta represent those that were defined as flexible residues during the docking process.


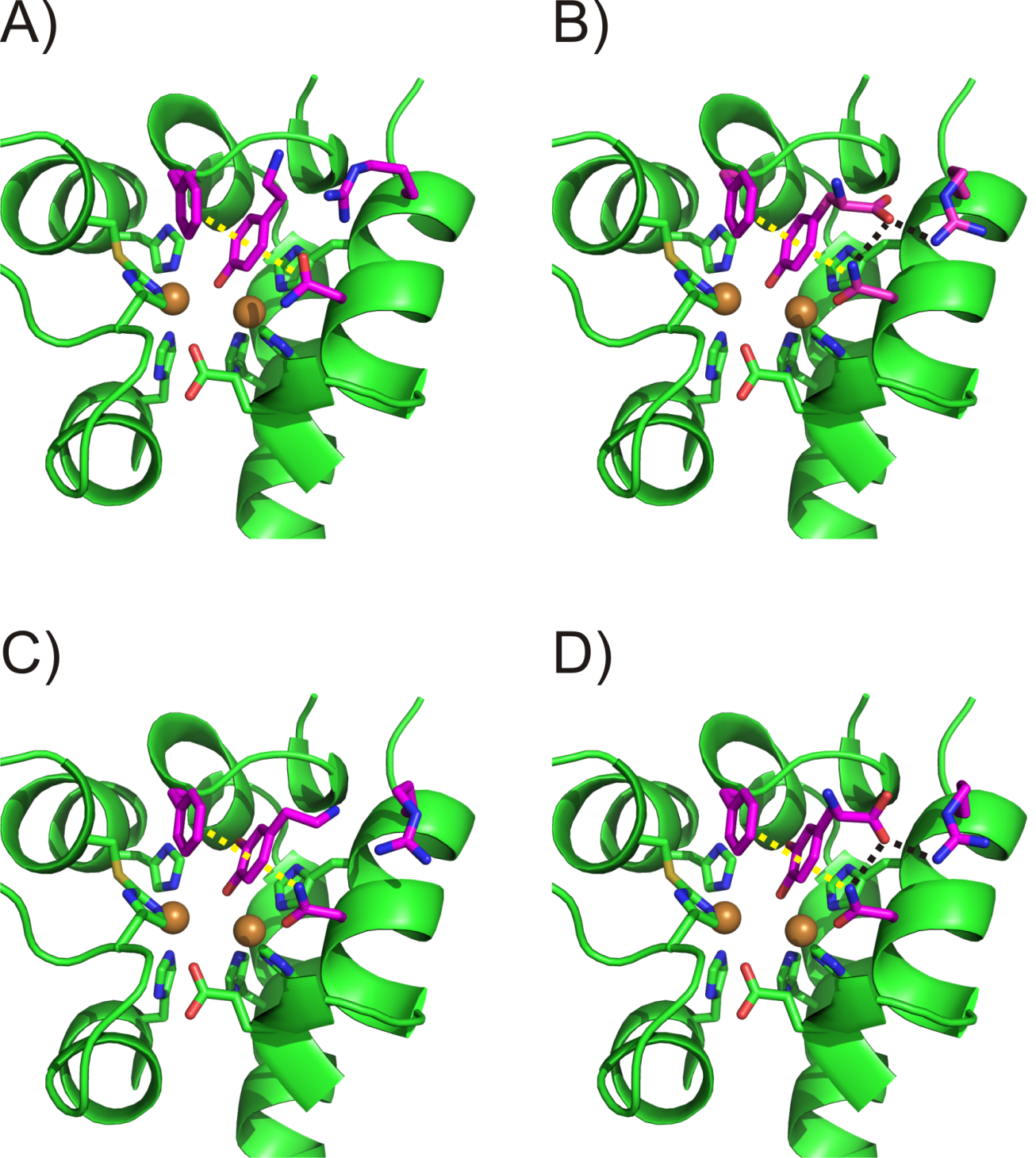


**Figure S20. Substrate binding to Leu244Arg according to docking**. Binding of A) tyramine, B) *L*-tyrosine, C) dopamine and D) *L*-DOPA. Each substrate is mainly stabilized by the Phe260-substrate-His243 π-stacking system (dashed yellow line). In the case of tyramine (A), the amine tail of the substrate is repelled by Arg244, leading to a slightly more vertical approach of the substrate towards the di-copper site. *L*-tyrosine (B) interacts strongly with Arg244 *via* its charged amide-tail (dashed black lines). In contrast to tyramine (A), the approach of dopamine (C) is not influenced by Arg244 as the latter is orientated away from the active site. The binding pose of *L*-DOPA (D) is almost identical to that of *L*-tyrosine (B). The residues colored in magenta represent those that were defined as flexible residues during the docking process.


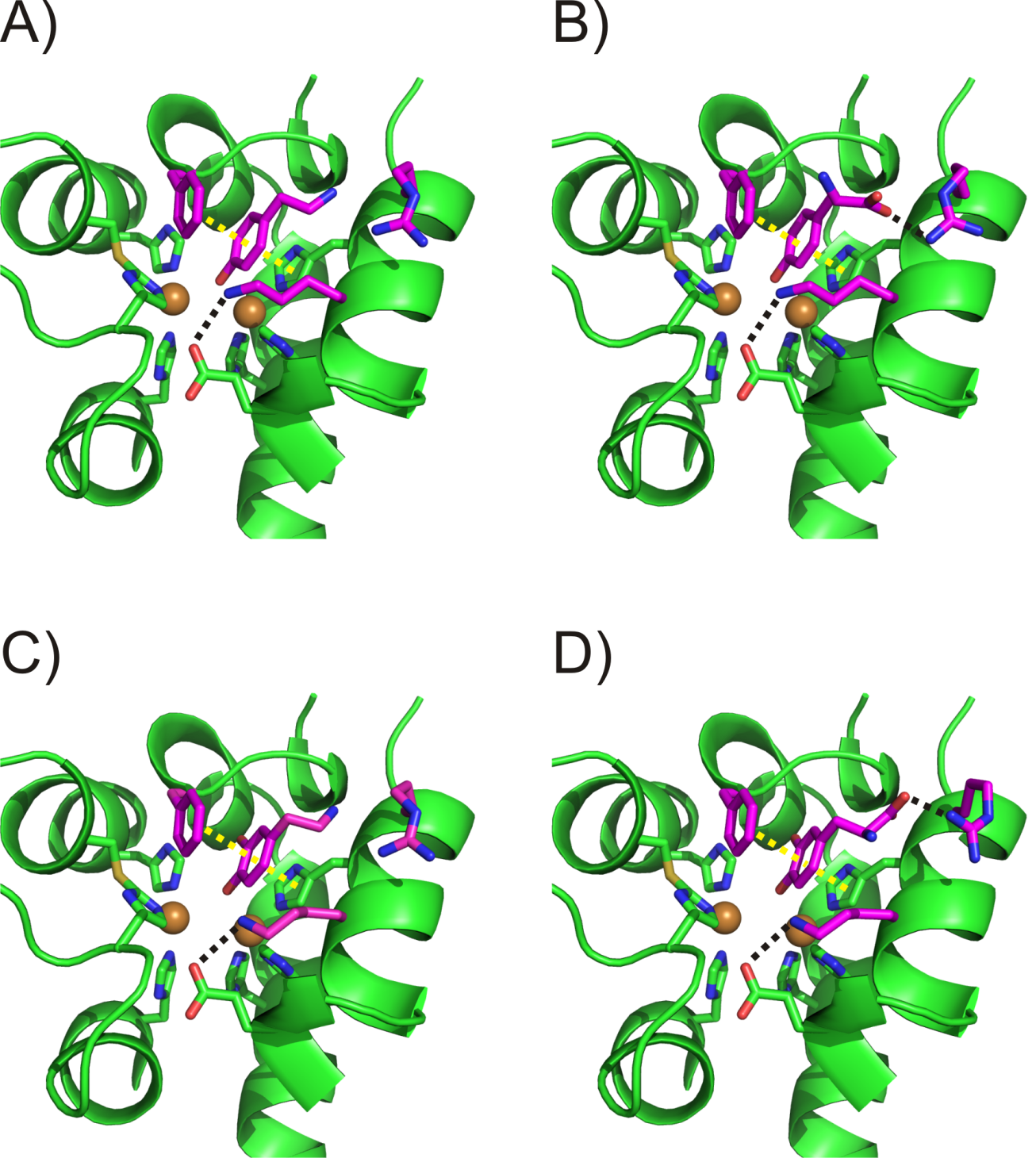


**Figure S21. Substrate binding to Asn240Lys/Leu244Arg according to docking**. Binding of A) tyramine, B) *L*-tyrosine, C) dopamine and D) *L*-DOPA. Each substrate is mainly stabilized by the Phe260-substrate-His243 π-stacking system (dashed yellow line). In each case Lys240 is able to electrostatically interact with Glu235 (dashed black lines). Tyramine and dopamine are correctly orientated within the active site as Arg244 is pointing towards the solvent region and therefore does not interfere with the substrates in a negative manner. *L*-tyrosine and *L*-DOPA are also ‘reasonably` positioned within the active site, however, they in addition exhibit electrostatic interactions with Arg244 *via* their carboxylate group (dashed black lines). The residues colored in magenta represent those that were defined as flexible residues during the docking process.


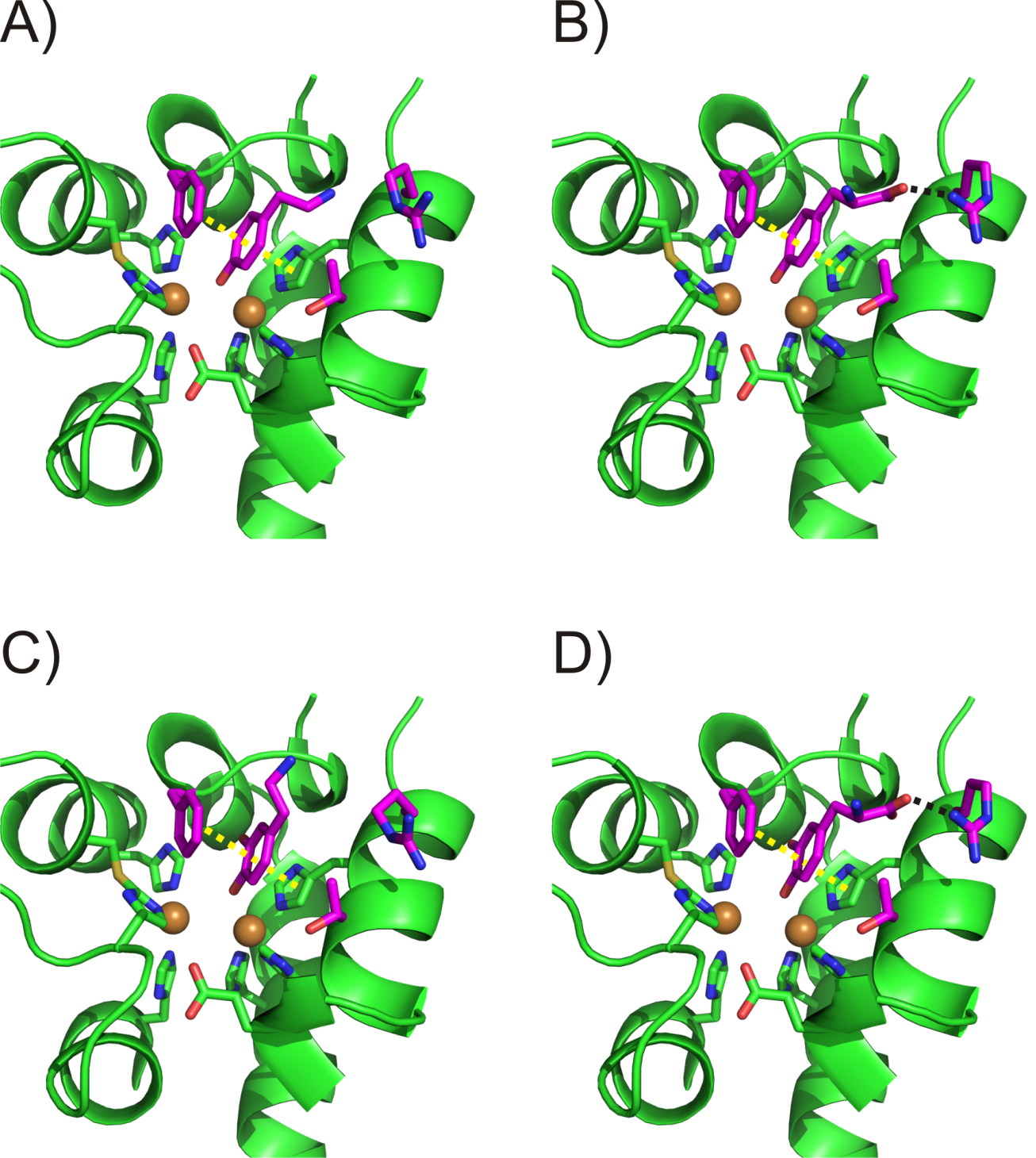


**Figure S22. Substrate binding to Asn240Thr/Leu244Arg according to docking**. Binding of A) tyramine, B) *L*-tyrosine, C) dopamine and D) *L*-DOPA. Each substrate is mainly stabilized by the Phe260-substrate-His243 π-stacking system (dashed yellow line). Therefore, all substrates are ‘reasonably’ positioned within the active site. Tyramine and dopamine repel Arg244, whereas *L*-tyrosine and *L*-DOPA interact electrostatically with the basic amino acid (dashed black lines). Thr240 is not involved in any interaction (only with the conserved water molecule which was not included in the docking and thus, is not depicted). The residues colored in magenta represent those that were defined as flexible residues during the docking process.


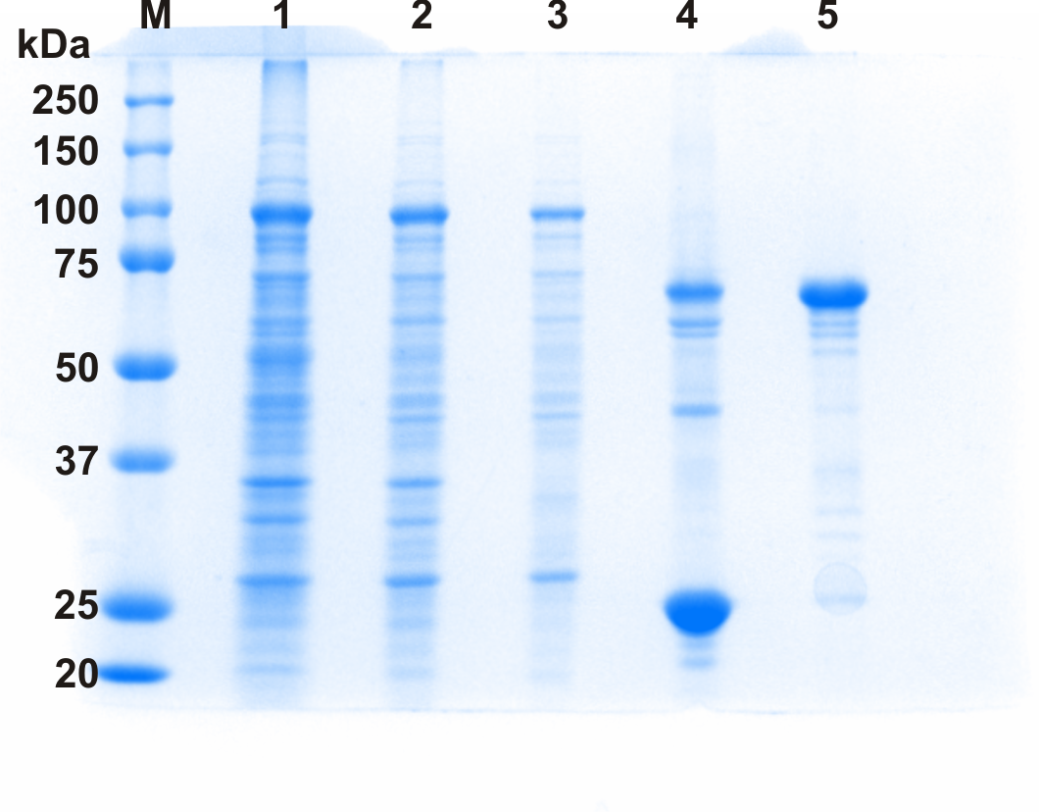


**Figure S23. Full-length gel of figure 3.** SDS-Page was performed using reducing conditions.


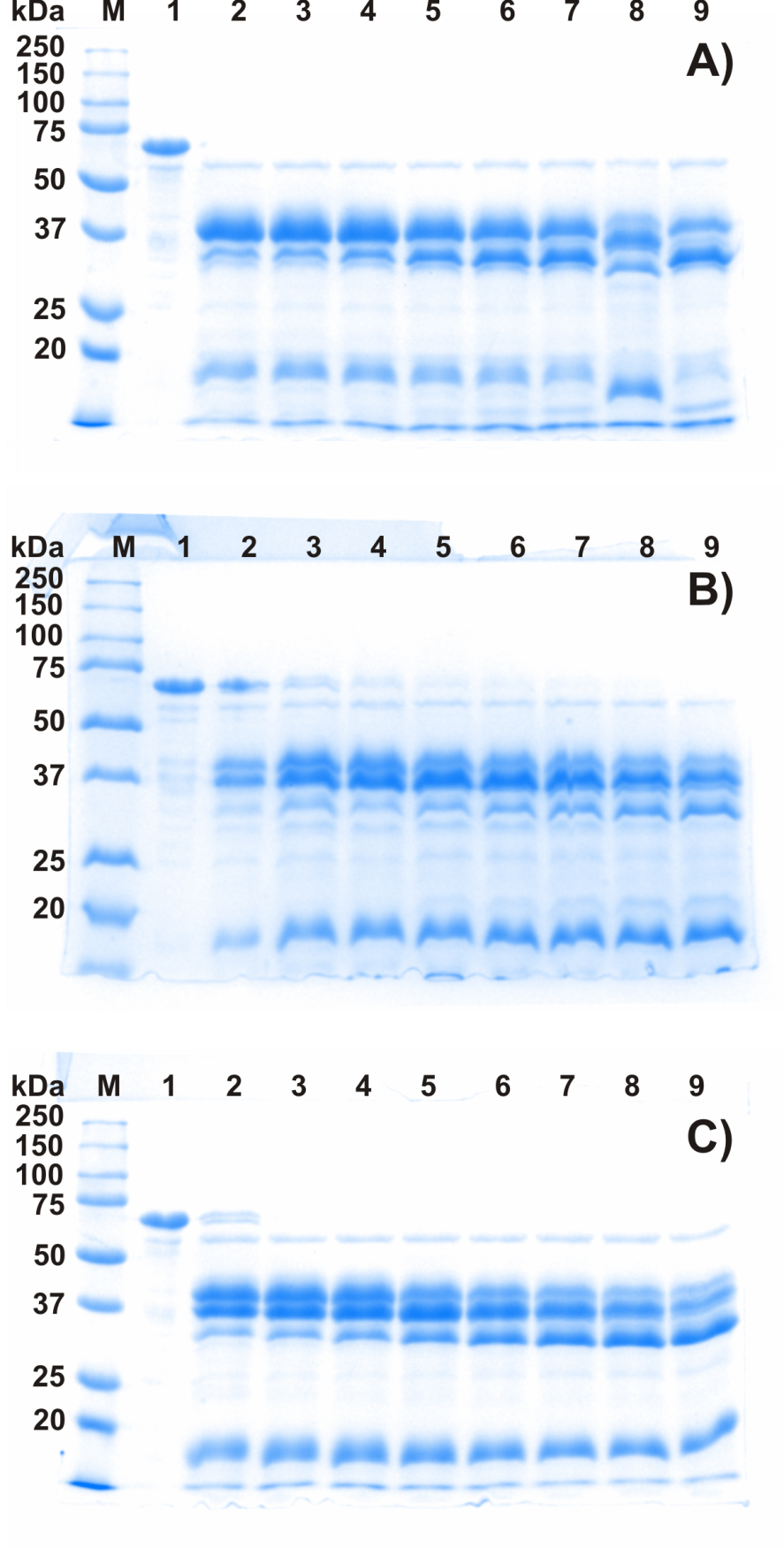


**Figure S24. Full-length gels of figure 4.** A: *jr*PPO1-wt with trypsin, B: *jr*PPO1-wt with nagarse, C: *jr*PPO1-wt with proteinase K after lane 1 = 0 minutes, lane 2 = 1 minutes, lane 3 = 2 minutes, lane 4 = 3 minutes, lane 5 = 5 minutes, lane 6 = 7 minutes, lane 7 = 10 minutes, lane 8 = 15 minutes, lane 9 = 20 minutes. Reducing SDS-PAGE was performed using 30 µg protein per lane. M = marker.


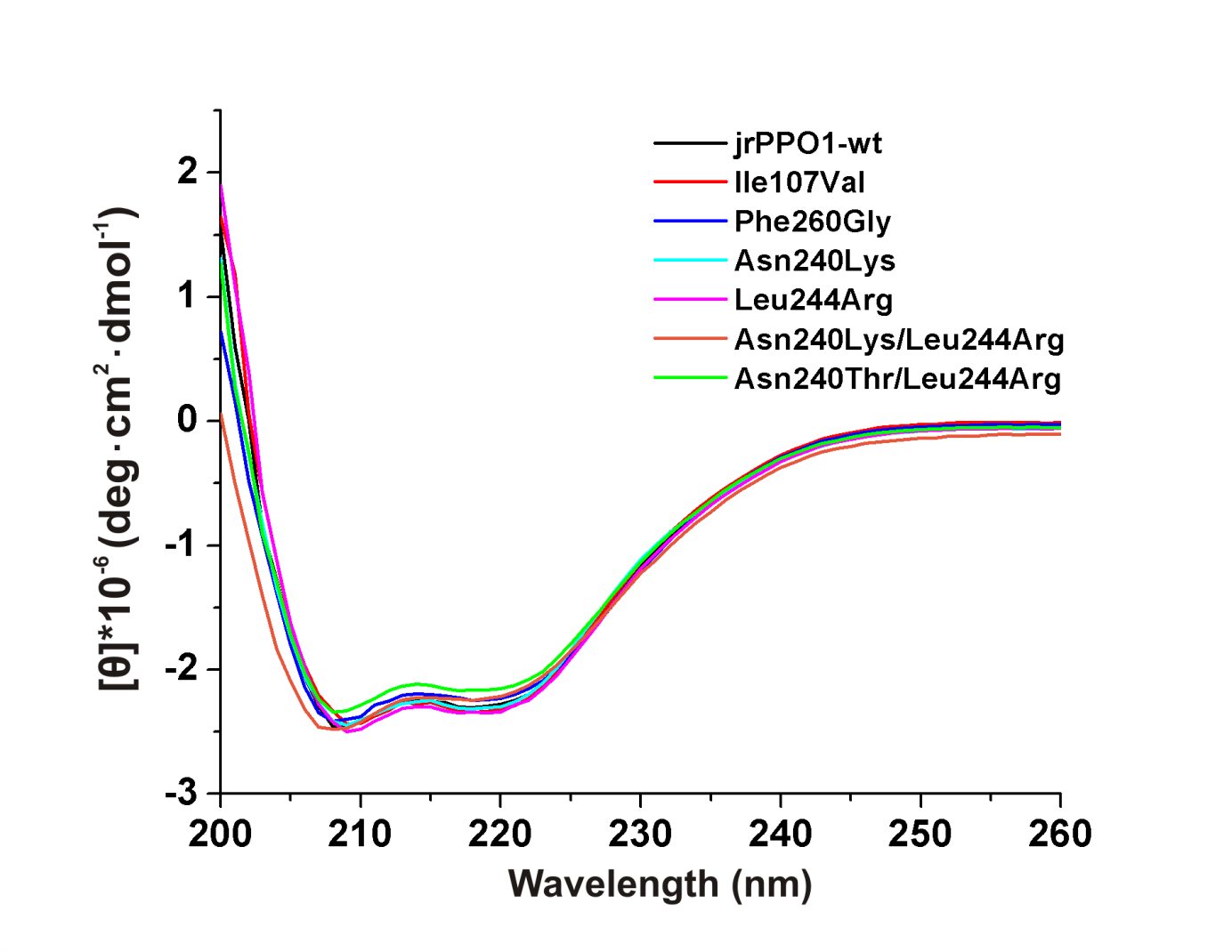


**Figure S25. CD-spectra of *jr*PPO1-wt, the five mutants investigated kinetically and the mutant Ile107Val.** Spectra were recorded on a Chirascan Plus spectropolarimeter (Applied Photophysics) purged sufficiently with N_2_ before use. A clear band at 208 nm and another band at 222 nm indicate the presence of alpha helices. All the samples were prepared at a protein concentration of 1 mg/ml in a buffer containing 50 mM sodium phosphate (pH 7.8) and 150 mM NaCl. Protein concentrations were determined spectrophotometrically. CD spectra were recorded at 20°C. The measurements were recorded from 200 to 260 nm using a precision quartz cuvette with a path length of 1 mm. The obtained spectra represent the average of five scans at a scan rate of 60 nm/min. The final spectra are presented after buffer subtraction.

**4. References**

1. Nillius, D., Jaenicke, E. & Decker, H. Switch between tyrosinase and catecholoxidase activity of scorpion hemocyanin by allosteric effectors. *FEBS Lett.* **582,** 749–754, doi:10.1016/j.febslet.2008.01.056 (2008).

2. Kampatsikas, I., Bijelic, A., Pretzler, M. & Rompel, A. Three recombinantly expressed apple tyrosinases suggest the amino acids responsible for mono- versus diphenolase activity in plant polyphenol oxidases. *Sci. Rep.* **7,** 8860, doi:10.1038/s41598-017-08097-5 (2017).

3. Katayama-Ikegami, A. *et al.* Recombinant expression, purification, and characterization of polyphenol oxidase 2 (*Vv*PPO2) from “Shine Muscat” (*Vitis labruscana* Bailey × *Vitis vinifera* L.). *Biosci. Biotechnol. Biochem.* **81,** 2330–2338, doi:10.1080/09168451.2017.1381017 (2017).

4. Derardja, A., Pretzler, M., Kampatsikas, I., Barkat, M. & Rompel, A. Purification and Characterization of Latent Polyphenol Oxidase from Apricot (*Prunus armeniaca L.*). *J. Agric. Food Chem.* **65,** 8203–8212, doi:10.1021/acs.jafc.7b03210 (2017).

5. Doğan, S. *et al.* Characterization and inhibition of *Rosmarinus officinalis* L. polyphenoloxidase. *Eur. Food Res. Technol.* **233,** 293–301, doi:10.1007/s00217-011-1504-y (2011).

6. Escobar M. A., Shilling A., Higgins P., Uratsu S. L. & Dandekar A. M. Characterization of Polyphenol Oxidase from Walnut. *J. Am. Soc. Hortic. Sci.* **133**, 852–858, doi:[10.21273/JASHS.133.6.852](https://doi.org/10.21273/JASHS.133.6.852) (2008).

7. Bijelic, A., Pretzler, M., Molitor, C., Zekiri, F. & Rompel, A. The Structure of a Plant Tyrosinase from Walnut Leaves Reveals the Importance of “Substrate-Guiding Residues” for Enzymatic Specificity. *Angew. Chemie Int. Ed.* **54,** 14677–14680 (2015).

8. Zekiri, F. *et al.* Purification and characterization of tyrosinase from walnut leaves (Juglans regia). *Phytochemistry* **101,** 5–15 (2014).

9. Wu, Y.-L., Pan, L.-P., Yu, S.-L. & Li, H.-H. Cloning, microbial expression and structure–activity relationship of polyphenol oxidases from *Camellia sinensis*. *J. Biotechnol.* **145**, 66–72, doi:10.1016/j.jbiotec.2009.10.008 (2010).

10. Sullivan, M. L., Hatfield, R. D., Thoma, S. L. & Samac, D. A. Cloning and Characterization of Red Clover Polyphenol Oxidase cDNAs and Expression of Active Protein in *Escherichia coli* and Transgenic Alfalfa. *Plant Physiol.* **136**, 3234–3244, doi:10.1104/ pp.104.047449 (2004).

11. Haruta, M. et al. Cloning Genomic DNA Encoding Apple Polyphenol Oxidase and Comparison of the Gene Product in *Escherichia coli* and in Apple. *Biosci. Biotechnol. Biochem.* **62**, 358–362, doi:10.1271/bbb.62.358 (1998).

12. Huang, C. *et al.* Two new polyphenol oxidase genes of tea plant (*Camellia sinensis*) respond differentially to the regurgitant of tea geometrid, *Ectropis obliqua*. *Int. J. Mol. Sci.* **19**, 1–17, doi:10.3390/ijms19082414 (2018).

13. Dirks-Hofmeister, M. E., Inlow, J. K. & Moerschbacher, B. M. Site-directed mutagenesis of a tetrameric dandelion polyphenol oxidase (PPO-6) reveals the site of subunit interaction. *Plant Mol. Biol.* **80**, 203–217, doi:10.1007/s11103-012-9943-9 (2012).

14. Prexler, S. M., Singh, R., Moerschbacher, B. M. & Dirks-Hofmeister, M. E. A specific amino acid residue in the catalytic site of dandelion polyphenol oxidases acts as ‘selector’ for substrate specificity. *Plant Mol. Biol.* **96**, 151-164, doi:10.1007/s11103-017-0686-5 (2017).

15. Kaintz, C. et al. Cloning and functional expression in *E. coli* of a polyphenol oxidase transcript from *Coreopsis grandiflora* involved in aurone formation. *FEBS Lett.* **588**, 3417–3426, doi:10.1016/j.febslet.2014.07.034 (2014).

16. Dirks-Hofmeister, M. E., Kolkenbrock, S. & Moerschbacher, B. M. Parameters That Enhance the Bacterial Expression of Active Plant Polyphenol Oxidases. *PLOS ONE* **8**, e77291, doi:10.1371/journal.pone.0077291 (2013).
